# Supplementary material for: Spatiotemporal Analysis of a Glycolytic Activity Gradient Linked to Mouse Embryo Mesoderm Development
Source: Dev Cell. 2017 Feb 27;40(4):331–341.e4. doi: 10.1016/j.devcel.2017.01.015 (PMC5337618; doi:10.1016/j.devcel.2017.01.015)
Supplement: Document S1. Figures S1–S5 and Table S1 [file mmc1.pdf]

Developmental Cell, Volume *40*

Supplemental Information

Spatiotemporal Analysis of a Glycolytic Activity Gradient Linked to Mouse Embryo Mesoderm Development

Vinay Bulusu, Nicole Prior, Marteinn T. Snaebjornsson, Andreas Kuehne, Katharina F. Sonnen, Jana Kress, Frank Stein, Carsten Schultz, Uwe Sauer, and Alexander Aulehla

Inventory of Supplemental Materials

Figure S1, related to Figure 1;
Figure S2, related to Figure 2;
Figure S3, related to Figure 3;
Figure S4, related to Figure 4;
Figure S5, related to Figure 5;
Table S1, related to Figure 1;
Movie S1 legend, related to Figure 5.

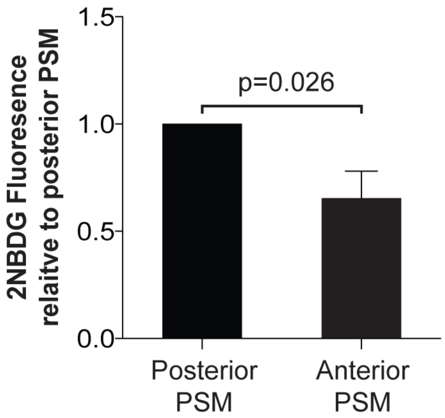

Figure S1 related to Figure 1.

Quantification of 2-NBDG fluorescence across the PSM reveals increased glucose uptake in posterior PSM after 21 hours *in vitro* culture. n=5, mean ± SDM.

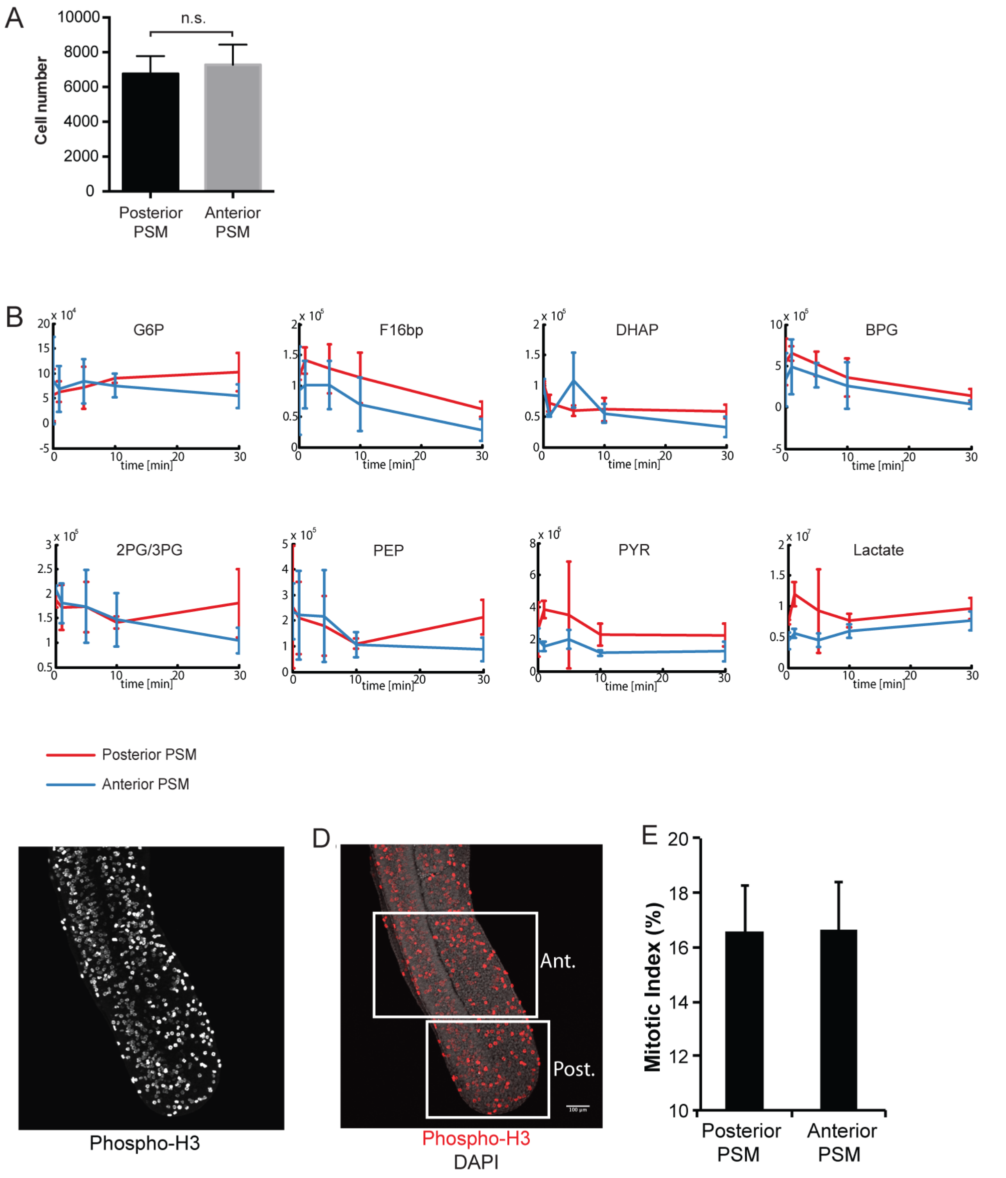

Figure S2 related to Figure 2.

Metabolite pool sizes remain constant between posterior and anterior PSM over time. (A) The number of cells counted in individual posterior and anterior PSM samples is not significantly different, n=14, mean ± SDM. (B) Summed raw intensities as a measure of intracellular pool size over the time course, n=3, mean ± SDM. Analysis of mitotic index reveals no significant difference between the anterior and posterior of mouse PSM. Embryonic tails (E10.5) were fixed directly after dissection, mitotic cells were detected using anti-Phospho-H3 antibodies (C) and counterstained with DAPI (merged in D). (E) Quantification of percentage of cells in M-phase in anterior compared to posterior PSM (N=5, mean ± SDM).

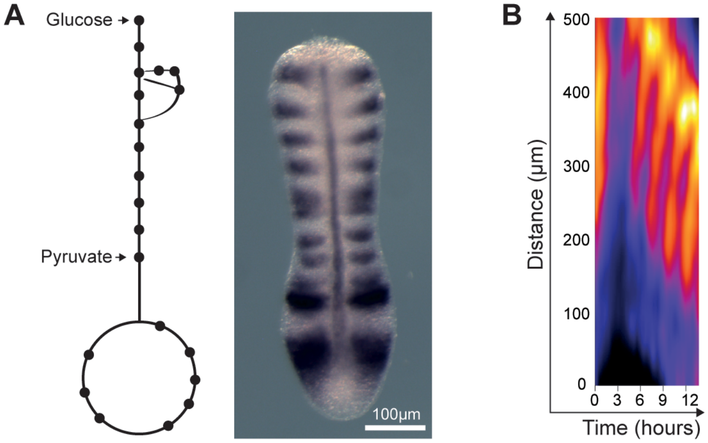

Figure S3 related to Figure 3.
Control experiment in which samples were cultured for 13 hours in medium containing 0.5mM Glucose and 10mM Pyruvate (in DMEM/F12).
(A) Whole-Mount mRNA ISH for *Lfng*, *Shh* and *Uncx4.1* following 13 hours culture is indistinguishable from results obtained in control medium (DMEM/F12=0.5mM Glucose), see Figure 3A
(B) Kymograph depicting activity of LuVeLu reporter activity within the PSM (distance from tail bud, posterior, in micrometer). Explants cultured in 0.5mM glucose + 10mM Pyruvate (in DMEM/F12) displayed regular cyclic activity of LuVeLu and segmentation throughout culture (indistinguishable from results obtained in control medium, DMEM/F12+0.5mM Glucose, see Figure 3C). n=3

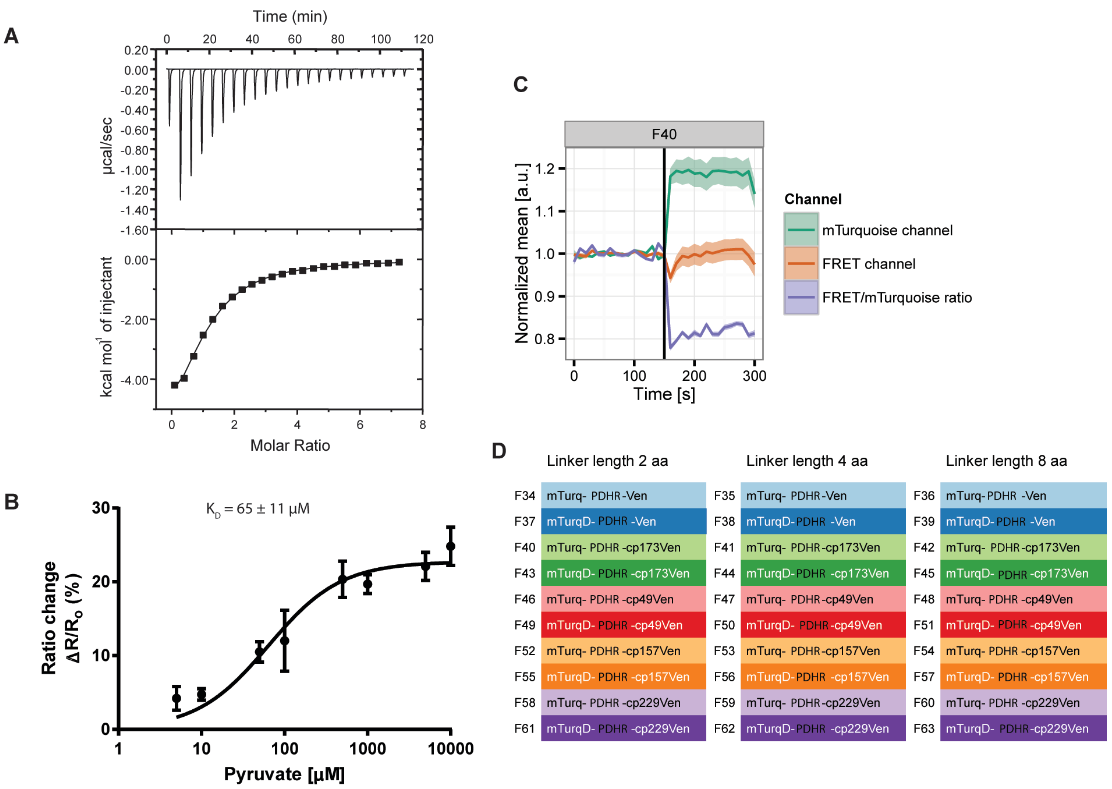

Figure S4 related to Figure 4.

(A) Pyruvate binding analysis with recombinant mTurquoise-PdhR-cp173-Venusd protein by isothermal titration calorimetry (ITC). Shown are the raw calorimetric signals obtained during the stepwise addition of pyruvate to the protein solution (60 µM) in the ITC calorimeter. The fit of the data using MicroCal Origin™ software package shows a K_d of 21 µM. (B) Pyruvate binding analysis with recombinant mTurquoise-PdhR-cp173-Venusd protein by in vitro FRET analysis. Data were fitted to a one site binding equation $Y = B_{max}*X/(K_D + [X])$, where X is the pyruvate concentration, B_{max} is the maximum specific binding and K_D is the dissociation constant using Graph pad Prism software. (C) Quantification of PYRATES-FRET signals before and after pyruvate addition in HeLa cells. Fluorescence quantification of donor and acceptor channels (mean ± SEM) before and after addition of 20mM Pyruvate (indicated by black bar). After addition of pyruvate, donor/mTurquoise fluorescence emission increases and acceptor/Venus fluorescence decreases. Overall, the Venus/mTurquoise ratio decreases upon addition of Pyruvate. The data is representative of 28 cell traces. (D) Library design for FRET optimization. Constructs with different mTurquoise (mTurq) and Venus (Ven) variants and different linker lengths (2, 4 or 8 amino acids (aa)) were used for FRET optimization.

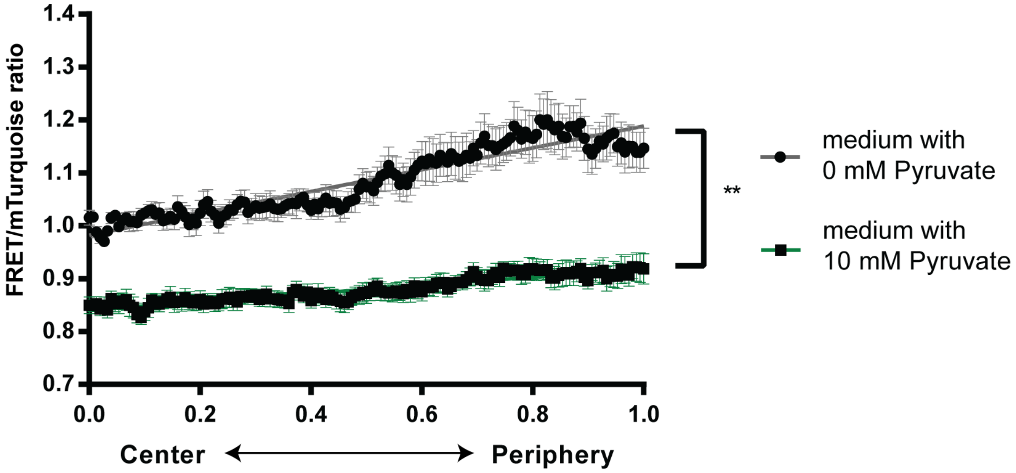

Figure S5 related to Figure 5.

FRET/mTurquoise ratio gradients from center to periphery of 2D *ex vivo* cultures (cultured for 18h) before (grey) and 2h after (green) addition of 10mM pyruvate to the control medium. Addition of exogenous pyruvate leads to a ~16% drop in mean FRET/mTurquoise ratio. The slopes of gradient before and after addition of exogenous pyruvate are significantly different. **$p < 0.001$, analysis of covariance (ANCOVA) test of linear regression lines. Data is mean ± SEM, n=10 samples from N=3 independent experiments.

Table S1 related to Figure 1. Mouse embryo *In Situ* mRNA hybridization screen for genes coding for glucose carrier and metabolic enzymes.
List of 113 genes whose expression was analyzed in 10.5dpc mouse embryos. The 21 genes that were found to have elevated expression in the posterior PSM are shown in bold, genes without detectable expression are shown in red.

Glycolysis

Glucose transporter 1 (GLUT1)
Glucose transporter 3 (GLUT3)
Hexokinase I (Hk1)
Hexokinase II (Hk2)
Hexokinase III (Hk3)
Glucokinase (Gck)
ADP dep. glucokinase (Adpgk)
Glucose phosphate isomerase 1 (Gpi1)
Phosphofruktokinase Platelet (Pfkp)
Phosphofruktokinase Liver (Pfkl)
Phosphofruktokinase Muscle (Pfkm)
Aldolase A (Aldoa)
Aldolase B (Aldob)
Aldolase C (Aldoc)
Triose phosphate isomerase 1 (Tpi1)
Glyceraldehyde-3P dehydrogenase (Gapdh)
Phosphoglycerate kinase 1 (Pgk1)
Phosphoglycerate kinase 2 (Pgk2)
Phosphoglycerate mutase 1 (Pgam1)
Phosphoglycerate mutase 2 (Pgam2)
Enolase 1 (Eno1)
Enolase 2 (Eno2)
Enolase 3 (Eno3)
Enolase 1B, retrotransposed (Eno1b)
Pyruvate kinase muscle (Pkm)
Lactate dehydrogenase A (Ldha)
Lactate dehydrogenase B (Ldhb)
Lactate dehydrogenase C (Ldhc)
Glucose transporter 2 (GLUT2)
Glucose transporter 4 (GLUT4)
Glucose transporter 5 (GLUT5)
Glucose transporter 6 (GLUT6)
GAPDH spermatogenic (Gapdhs)
Pyruvate kinase liver & red blood cell (Pklr)

Gluconeogenesis

Fructose bisphosphatase 1 (Fbp1)
Fructose bisphosphatase 2 (Fbp2)
Phosphoenolpyruvate carboxykinase 1 (Pck1)
Phosphoenolpyruvate carboxykinase 2 (Pck2)

TCA, PDH

Pyruvate dehydrogenase component X (Pdhx)
Pyruvate dehydrogenase E1 α1 (Pdha1)
Pyruvate dehydrogenase E1 α2 (Pdha2)
Pyruvate dehydrogenase β (Pdhb)
Pyruvate Carboxylase (Pcx)
Dihydrolipoamide S-acetyltransf. (Dlat)
Dihydrolipoamide dehydrogenase (Dld)
Citrate synthase (Cs)
Citrate synthase like (Csl)
ATP citrate lyase (Acly)
Aconitase 1 (Aco1)
Aconitase 2, mitochondrial (Aco2)
Isocitrate dehydrogenase 1 (Idh1)
Isocitrate dehydrogenase 2 (Idh2)
Isocitrate dehydrogenase 3β (Idh3b)
Isocitrate dehydrogenase 3γ (Idh3g)
Oxoglutarate dehydrogenase (Ogdh)
Oxoglutarate dehydrogenase like (Ogdhl)
Dihydrolipoamide S-succinyltransferase (Dlst)
Succinate-CoA ligase α, GDP (Suclg1)
Succinate-CoA ligase β, GDP (Suclg2)
Succinate-CoA ligase β, ADP (Sucla2)
Succinate dehydrogenase A (Sdha)
Succinate dehydrogenase B (Sdhb)
Succinate dehydrogenase C (Sdhc)
Fumarate hydratase 1 (Fh1)
Malate dehydrogenase 1 (Mdh1)
Malate dehydrogenase 2 (Mdh2)
Isocitrate dehydrogenase 3α (Idh3a)

Acetate metabolism

Aldehyde dehydrogenase 1A3 (Aldh1a3)
Aldehyde dehydrogenase 1B1 (Aldh1b1)
Aldehyde dehydrogenase 2, mit. (Aldh2)
Aldehyde dehydrogenase 3A1 (Aldh3a1)
Aldehyde dehydrogenase 3A2 (Aldh3a2)
Aldehyde dehydrogenase 3B1 (Aldh3b1)
Aldehyde dehydrogenase 7A1 (Aldh7a1)
Aldehyde dehydrogenase 9A1 (Aldh9a1)
Acyl-CoA synthase 1 (Acss1)
Acyl-CoA synthase 2 (Acss2)

Pentose phosphate pathway

G6P dehydrogenase X (G6pdx)
H6P dehydrogenase (H6pd)
6-phosphate gluconolactonase (Pgls)
Phosphogluconate dehydrog. (Pgd)
Ribulose-5P-3-epimerase (Rpe)
Ribose-5P isomerase A (Rpla)
Transketolase (Tkt)
Transketolase like 1 (Tktl1)
Transketolase like 2 (Tktl2)
Transaldolase 1 (Taldo1)
Ribokinase (Rbks)
Phosphoglucomutase 1 (Pgm1)
Phosphoglucomutase 2 (Pgm2)
Phosphoribosyl pyrophatase synthetase 1 (Prps1)
Phosphoribosyl pyrophatase synthetase 2 (Prps2)
2-deoxyribose-5P aldolase homolog (Dera)
G6P dehydrogenase 2 (G6pd2)

Galactose metabolism

Aldo-keto reductase 1B3 (Akr1b3)
Aldo-keto reductase 1B7 (Akr1b7)
Aldo-keto reductase 1B8 (Akr1b8)
Galactosidase α (Gla)
Galactose mutarotase (Galm)
Galaktokinase 2 (Galk2)
Galactose-1P uridyl transf. (Galt)
UDP-glucose pyrophosphorylase 2 (Ugp2)
Glucose-6P, catalytic (G6pc)
Lactase (Lct)
Galactosidase β1 (Glb1)
Glucosidase α, acid (Gaa)
Glucosidase α, neutral C (Ganc)
UDP-Gal:βGlcNac β 1,4-Galactosyltransf. 1 (B4galt1)
UDP-Gal:βGlcNac β 1,4-Galactosyltransf. 2 (B4galt2)
Maltase-Glucoamylase (Mgam)
Galactokinase 1 (Galk1)
Lactalbumin α (Lalba)
Galactose-4-epimerase, UDP (Gale)

Metabolic pathway:	Total genes screened:	Detected in the embryo:	Detected in the PSM:	Glycolytic PSM expresion Pattern:
All pathways	113 genes	99 genes	79 genes	21/79 (27%)
Glycolysis	34 genes	28 genes	24 genes	17/24 (71%)
TCA, PDH	29 genes	28 genes	22 genes	3/22 (14%)
PPP	17 genes	16 genes	13 genes	0/13 (0%)
Acetate metabolism	10 genes	10 genes	6 genes	0/6 (0%)
Gluconeogenesis	4 genes	4 genes	1 gene	0/1 (0%)
Galactose metabolism	19 genes	15 genes	14 genes	1/14 (0.07%)

Movie S1 legend, related to Figure 5. Real-time imaging of FRET-pyruvate sensor reporter PYRATES in 2-D *ex vivo* mesoderm segmentation assay. Tail bud mesoderm derived from a 10.5dpc mouse embryo heterozygous for the PYRATES transgene was used for 2-D *ex vivo* culture. Real-time imaging reveals the formation of a FRET ratio gradient from center to periphery during the course of *ex vivo* culture, which corresponds to cells with posterior and anterior PSM identify, respectively. A quantification of this movie is shown as kymograph in Fig5F.
