# Supplementary figures and images for: Spatiotemporal Analysis of a Glycolytic Activity Gradient Linked to Mouse Embryo Mesoderm Development
Source: Dev Cell. 2017 Feb 27;40(4):331–341.e4. doi: 10.1016/j.devcel.2017.01.015 (PMC5337618; doi:10.1016/j.devcel.2017.01.015)

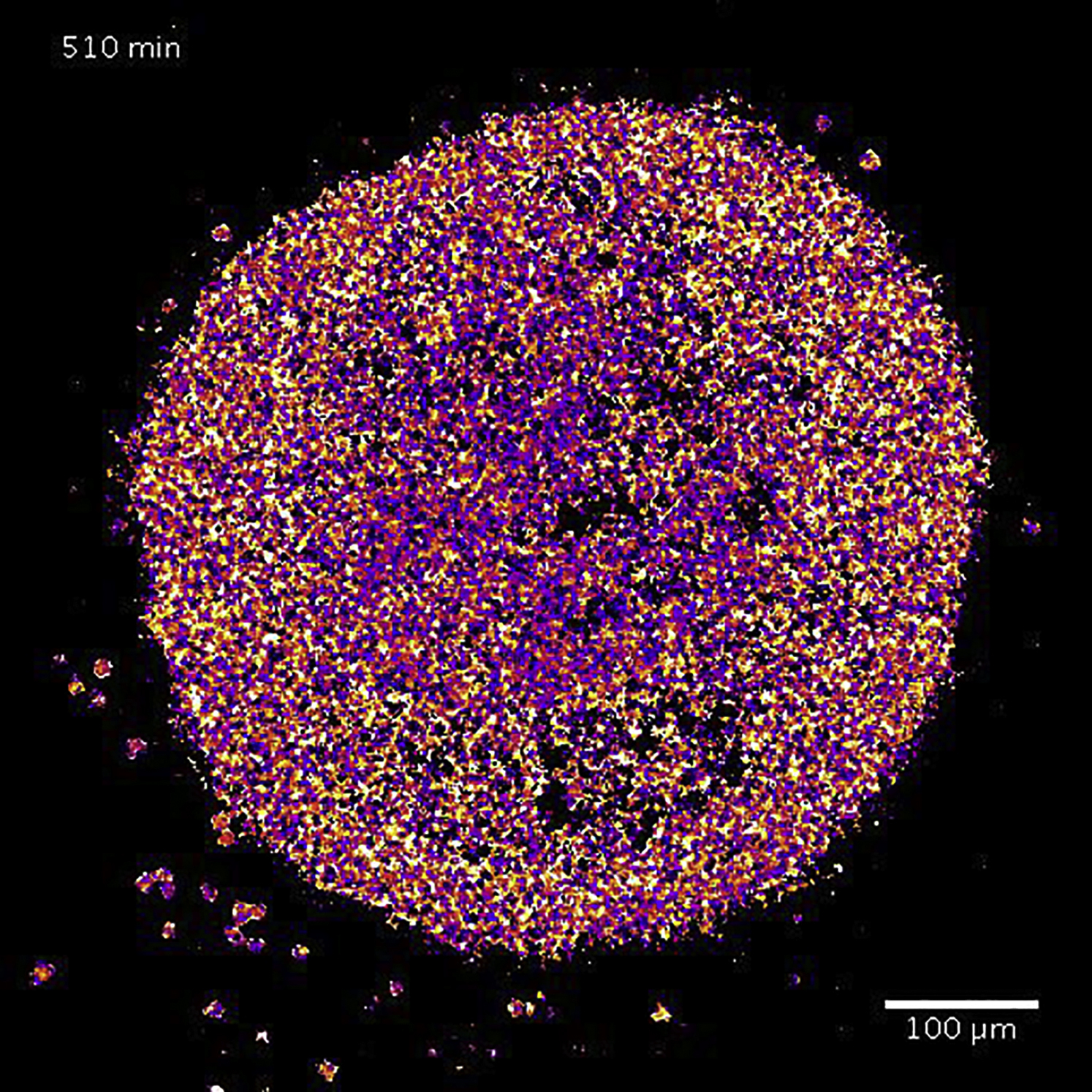

Supplement: Movie S1. Real-Time Imaging of FRET-Pyruvate Sensor Reporter PYRATES in 2D Ex Vivo Mesoderm Segmentation Assay, Related to Figure 5 [file mmc2.jpg]
