# Supplementary material for: Spatiotemporal Analysis of a Glycolytic Activity Gradient Linked to Mouse Embryo Mesoderm Development
Source: Dev Cell. 2017 Feb 27;40(4):331–341.e4. doi: 10.1016/j.devcel.2017.01.015 (PMC5337618; doi:10.1016/j.devcel.2017.01.015)
Supplement: Document S2. Article plus Supplemental Information [file mmc3.pdf]

Developmental Cell

Article

Spatiotemporal Analysis of a Glycolytic Activity Gradient Linked to Mouse Embryo Mesoderm Development

Graphical Abstract

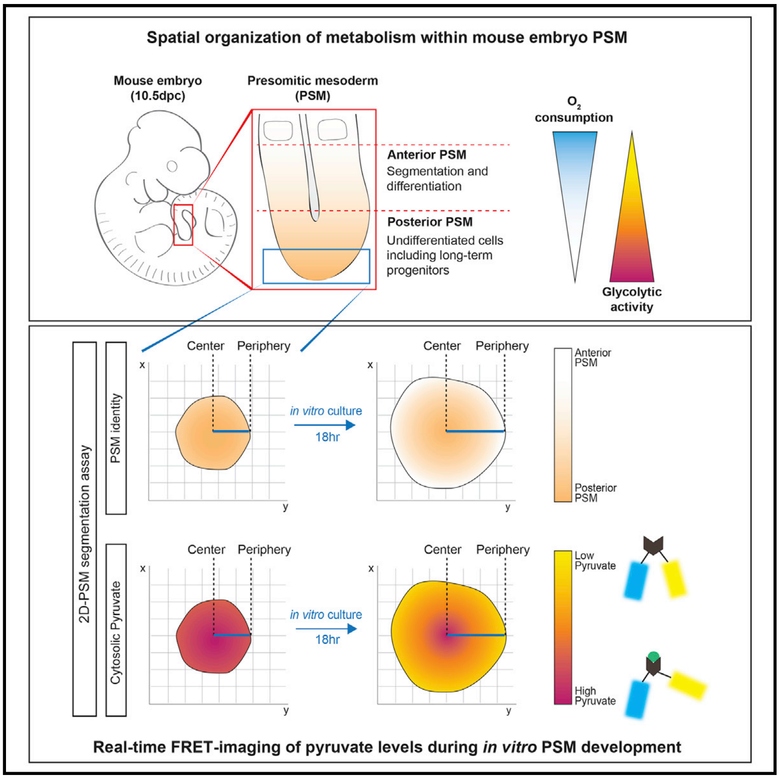

Highlights

- Identification of glycolytic activity gradient in mouse presomitic mesoderm
- Development of a pyruvate FRET-reporter mouse model
- Real-time imaging reveals pyruvate gradient dynamics
- Metabolic state is linked to presomitic mesoderm cell differentiation

Authors

Vinay Bulusu, Nicole Prior, Marteinn T. Snaebjornsson, ..., Carsten Schultz, Uwe Sauer, Alexander Aulehla

Correspondence

aulehla@embl.de

In Brief

Bulusu, Prior, Snaebjoernsson et al. identify a glycolytic activity gradient in the mouse embryo during presomitic mesoderm (PSM) development. Using a FRET-pyruvate sensor mouse model for real-time imaging of metabolite levels, they find that spatial metabolic differences are functionally relevant and intrinsically linked to PSM cell differentiation and development.

Bulusu et al., 2017, Developmental Cell *40*, 331–341
February 27, 2017 © 2017 The Authors. Published by Elsevier Inc.
http://dx.doi.org/10.1016/j.devcel.2017.01.015

CellPress

Developmental Cell
Article

Spatiotemporal Analysis of a Glycolytic Activity Gradient Linked to Mouse Embryo Mesoderm Development

Vinay Bulusu,[1,2,4] Nicole Prior,[1,4] Marteinn T. Snaebjornsson,[1,4] Andreas Kuehne,[3] Katharina F. Sonnen,[1] Jana Kress,[1] Frank Stein,[2] Carsten Schultz,[2] Uwe Sauer,[3] and Alexander Aulehla[1,5,*]
[1]Developmental Biology Unit
[2]Cell Biology & Biophysics Unit
European Molecular Biology Laboratory (EMBL), 69117 Heidelberg, Germany
[3]Institute of Molecular Systems Biology, ETH Zurich, 8093 Zurich, Switzerland
[4]Co-first author
[5]Lead Contact
*Correspondence: aulehla@embl.de
http://dx.doi.org/10.1016/j.devcel.2017.01.015

SUMMARY

How metabolism is rewired during embryonic development is still largely unknown, as it remains a major technical challenge to resolve metabolic activities or metabolite levels with spatiotemporal resolution. Here, we investigated metabolic changes during development of organogenesis-stage mouse embryos, focusing on the presomitic mesoderm (PSM). We measured glycolytic labeling kinetics from ^{13}C-glucose tracing experiments and detected elevated glycolysis in the posterior, more undifferentiated PSM. We found evidence that the spatial metabolic differences are functionally relevant during PSM development. To enable real-time quantification of a glycolytic metabolite with spatiotemporal resolution, we generated a pyruvate FRET-sensor reporter mouse line. We revealed dynamic changes in cytosolic pyruvate levels as cells transit toward a more anterior PSM state. Combined, our approach identifies a gradient of glycolytic activity across the PSM, and we provide evidence that these spatiotemporal metabolic changes are intrinsically linked to PSM development and differentiation.

INTRODUCTION

The metabolic changes underlying cellular growth, differentiation, and transformation have been extensively addressed in cancer biology and during stem cell differentiation (Agathocleous and Harris, 2013; Vander Heiden et al., 2009). In contrast, very little is known about the metabolic state in the context of mammalian embryonic development, particularly in organogenesis-stage mammalian embryos. With the onset of placental function occurring at day 9 of mouse development (Watson and Cross, 2005), embryos switch from anaerobic glycolysis to a state relying considerably on oxidative respiration (Shepard et al., 1997). However, these previous global and static measurements were unable to resolve dynamic changes in metabolic activities or metabolite levels with spatiotemporal resolution.

To address this challenge, we used mouse embryo mesoderm segmentation as a model. Mesoderm segmentation occurs within the presomitic mesoderm (PSM), which consists of undifferentiated mesenchymal cells located at the posterior end of vertebrate embryos. Cells from the posterior PSM, which also contains long-term progenitors (Cambray and Wilson, 2002), gradually change their transcriptional program and transit toward a more differentiated state in the anterior PSM. Here, a group of cells is periodically partitioned to form somites, prevertebrae, which subsequently will further differentiate into various tissues such as bone, cartilage, and striated muscles.

Numerous studies have provided clear evidence that somite formation and PSM patterning is controlled by several major signaling pathways, such as the Wnt (Aulehla et al., 2003, 2008; Bajard et al., 2014), fibroblast growth factor (FGF), and retinoic acid pathways (Diez del Corral et al., 2003; Dubrulle et al., 2001; Moreno and Kintner, 2004; Sawada et al., 2001). These signaling pathways show a graded activity within the PSM and have been shown to control PSM differentiation (Hubaud and Pourquie, 2014). In addition, several of these signaling pathways, including the Notch-signaling pathway, show oscillatory activity in PSM cells and are part of the segmentation clock, which controls the timing of periodic somite formation (Hubaud and Pourquie, 2014; Krol et al., 2011).

In contrast to the insight into the signaling network underlying PSM development, little is known about how metabolic activities are spatially and temporally distributed during mouse PSM patterning and differentiation. An in situ mRNA screen in mouse embryos at 14.5 days post coitum (dpc) revealed that genes coding for glycolytic enzymes show tissue-specific expression patterns (Cankaya et al., 2007). However, any dynamic or functional metabolic state analysis was not performed. Similarly, microarray mRNA profiling performed in zebrafish embryos indicated graded enzyme expression and metabolic activities along the PSM (Ozbudak et al., 2010). Whether such metabolic activities are also spatially organized in mammalian embryos during segmentation and whether metabolic dynamics

This is an open access article under the CC BY license (http://creativecommons.org/licenses/by/4.0/).

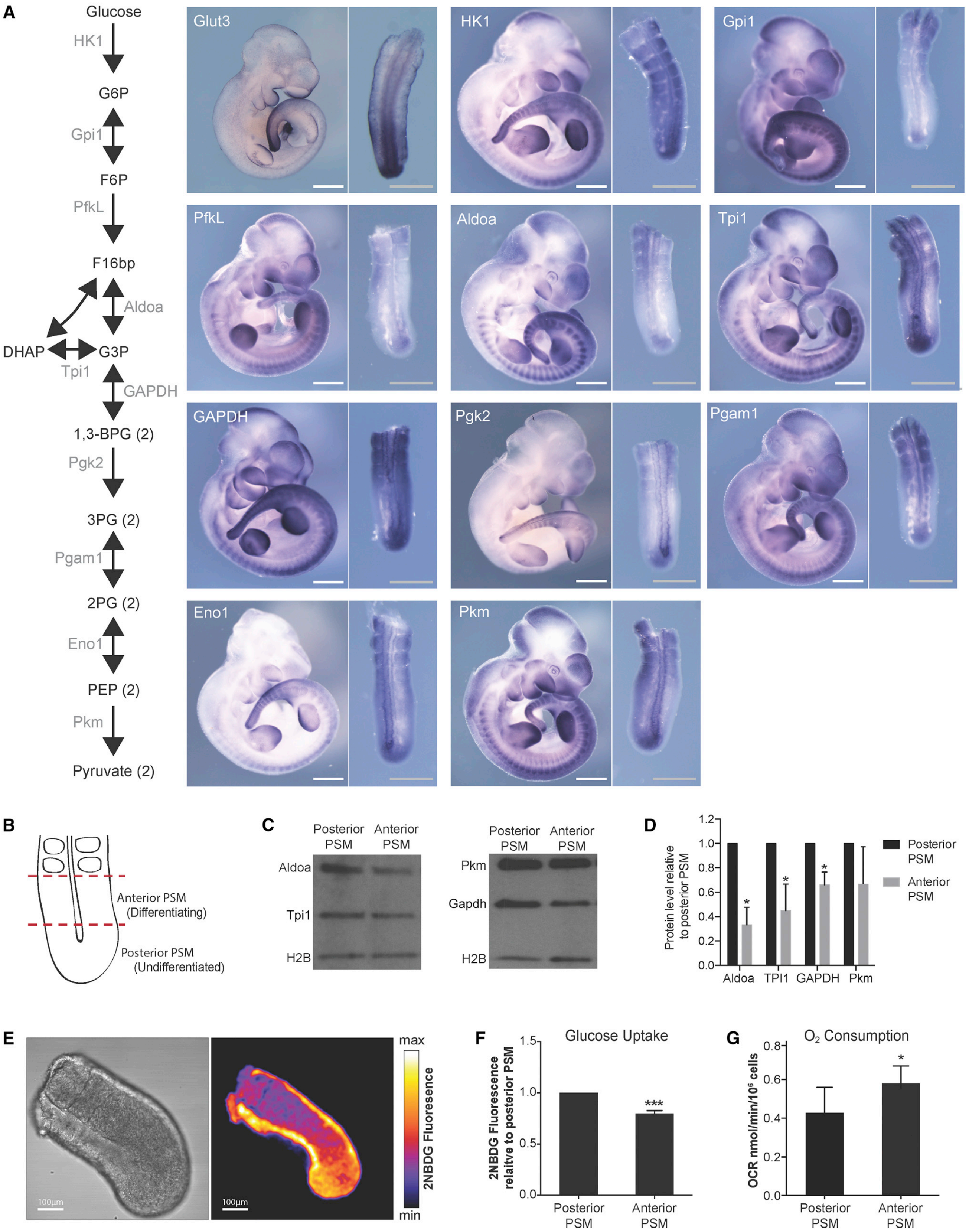

(legend on next page)

are intrinsically linked to differentiation has not been investigated thus far.

In this work, we developed novel experimental approaches with the goal of addressing these questions in the context of mammalian embryonic mesoderm development.

RESULTS

Expression Pattern Analysis of Metabolic Enzymes Indicates Region-Specific Metabolic Profiles

To address the metabolic state in embryonic development and in particular during PSM patterning, we initially performed an mRNA expression screen for 113 genes coding for glucose carriers and metabolic enzymes in mouse embryos at 10.5 dpc. While many metabolic enzymes expectedly exhibited high and ubiquitous expression throughout the embryo (Table S1 and data not shown), the majority of glycolytic genes (17 out of 24 expressed in the PSM) showed higher expression in the posterior, undifferentiated PSM and lower expression toward the anterior PSM (Figure 1A). This expression gradient in the PSM was not observed for pentose phosphate pathway genes (0/13) and for only 3 out of 22 tricarboxylic acid (TCA) cycle genes (Table S1). We confirmed that differential mRNA expression of glycolytic enzymes was also translated to the protein level and found increased protein levels of aldolase A (Aldoa), triose phosphate isomerase (Tpi), and glyceraldehyde-3-phosphate dehydrogenase (Gapdh) in the posterior, undifferentiated PSM (Figures 1B–1D).

The differential gene and protein expression of glycolytic enzymes along the PSM raised the question of whether also at the functional level glycolysis operates differently across the PSM. As indirect evidence, we indeed found increased uptake of the fluorescent glucose analog 2-NBDG in the posterior PSM (Figures 1E, 1F, and S1). In turn, the basal oxygen consumption rate was enhanced in the anterior relative to the posterior PSM (Figure 1G). Combined, these results provided first evidence for differences in metabolic state across the mouse PSM.

Kinetic ^{13}C-Isotope Flux Analysis Reveals a Glycolytic Activity Gradient along the PSM

For a direct quantification of glycolytic activity we performed dynamic ^{13}C tracing experiments in mouse embryonic tissues. We cultured anterior and posterior PSM in media containing uniformly labeled -^{13}C glucose and analyzed the label propagation in glycolytic metabolites at several time points within the first 30 min after addition of fully labeled -^{13}C glucose. A major analytical challenge was the low number of cells per embryo sample (i.e., ~7,000 cells/PSM fragment), which we overcame by pooling PSM fragments (20 PSM fragments per time point) and by using highly sensitive liquid chromatography-tandem mass spectrometry (Figure 2A). The rate of ^{13}C incorporation into all detected glycolytic intermediates, such as glucose-6-phosphate (G6P), fructose 1,6-bisphosphate (F16bP), and phosphoenolpyruvic acid (PEP), was higher in posterior PSM (solid lines) compared with anterior PSM (dashed lines) (Figure 2B). Since the metabolite pool sizes and cell numbers were similar in anterior and posterior PSM fragments (Figures S2A and S2B), the faster label propagation directly demonstrated higher glycolytic flux in the posterior PSM relative to the anterior PSM.

The finding of glycolytic differences within the PSM raised the immediate question of whether these primarily reflect differences at the level of basic cellular functions, such as an altered rate of cell proliferation between cells in the anterior and posterior PSM. We therefore analyzed cell proliferation across PSM and did not find, in agreement with previous findings (Venters et al., 2008), any significant differences in mitotic index between anterior and posterior PSM (Figures S2C–S2E). Hence, we conclude that increased glycolysis in the posterior PSM is not readily accounted for by differences of proliferative activity.

Bypassing Glycolysis Causes a Region-Specific, Posterior PSM Phenotype

Next, we asked whether the detected differences in glycolytic flux are, in fact, physiologically significant. We reasoned that if this was the case, regional differences regarding the requirement for ongoing glycolysis should exist. We therefore tested the consequence of bypassing glycolytic activity in anterior and posterior PSM regions. To this end, we compared PSM explants cultured in control conditions (Lauschke et al., 2013), consisting of a nutrient-rich, serum-free medium (DMEM/F12 without glucose and pyruvate), supplemented with 0.5 mM glucose, to samples cultured in the identical medium except that glucose was replaced by pyruvate (10 mM, "pyruvate condition"). While

Figure 1. In Situ Hybridization Screen of Glucose Carrier and Glycolytic Enzymes in 10.5-dpc Mouse Embryo

(A) In situ hybridization (ISH) for mRNA expression of glucose transporters and glycolytic enzymes in 10.5-dpc embryo. Higher expression of glucose transporter 3 (Glut3) and at least one paralog each for all ten glycolytic enzymatic reactions is enriched in posterior compared with anterior PSM, in addition to other sites of expression (branchial arches, limb buds, somites). Right-hand images show a magnified dorsal view of PSM. HK1, hexokinase 1; Gpi1, glucose-6-phosphate isomerase 1; PfkL, phosphofructokinase L; Aldoa, aldolase A; Tpi1, triose phosphate isomerase 1; Gapdh, glyceraldehyde-3-phosphate dehydrogenase; Pgk2, phosphoglycerate kinase 2; Pgam1, phosphoglycerate mutase 1; Eno1, enolase 1; Pkm, pyruvate kinase M. n = 5. White scale bars, 500 μm; gray scale bars, 200 μm.

(B) Schematic of an embryonic tissue explant illustrating the regions considered for further analysis, i.e., posterior PSM and anterior PSM.

(C) Immunoblot analysis of glycolytic enzymes in posterior and anterior PSM. Aldoa, aldolase A; Tpi1, triose phosphate isomerase 1; Pkm, pyruvate kinase M; Gapdh, glyceraldehyde-3-phosphate dehydrogenase; H2B, histone H2B.

(D) Relative normalized band intensities of glycolytic enzymes. Band intensities were normalized to histone H2B and plotted relative to posterior PSM, which was set to 1. Results are presented as mean ± SD, n = 3. Statistical significance is displayed as $^{*}p < 0.05$.

(E) 2-NBDG staining of an embryonic explant as an indirect readout for glucose uptake. Left panel shows the bright-field channel and the right panel shows 2-NBDG fluorescence.

(F) Quantification of 2-NBDG fluorescence across the PSM. 2-NBDG intensities in posterior and anterior PSM were measured and plotted relative to posterior PSM. Results are presented as mean ± SD, n = 3. Student's t test (paired two-tailed distribution) was used to calculate p values. Statistical significance is displayed as $^{***}p < 0.001$.

(G) Basal oxygen consumption rate (OCR) measurements of anterior and posterior PSM explants. Results are presented as mean ± SD, n = 3. Student's t test (paired two-tailed distribution) was used to calculate p values. Statistical significance is displayed as $^{*}p < 0.05$.

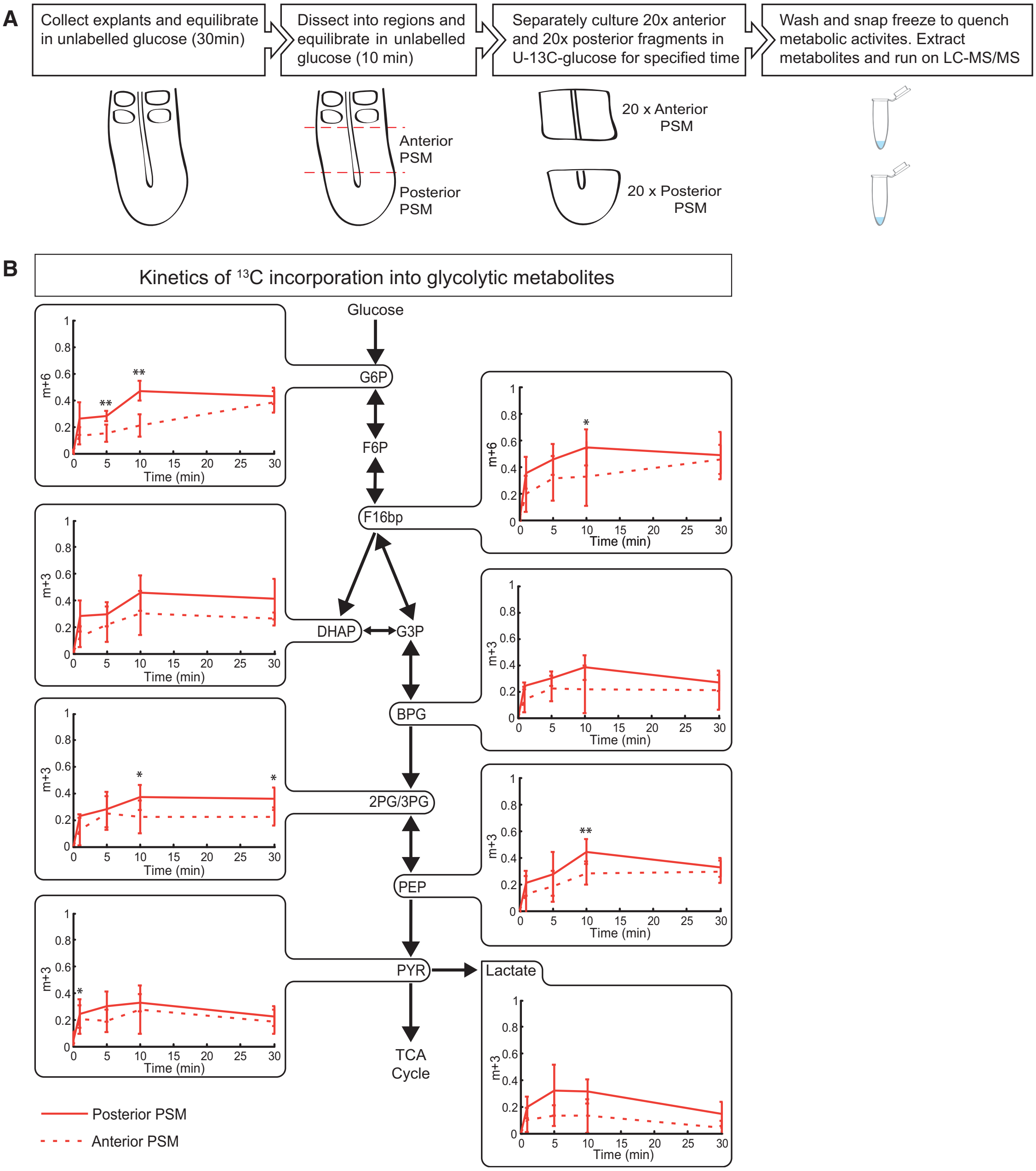

Figure 2. Glycolytic Labeling Kinetics from ^{13}C-Isotope Tracing in Mouse Embryonic Explants Shows Increased Glycolytic Flux in the Posterior PSM

(A) Schematic of the experimental setup for kinetic U-^{13}C glucose labeling of mouse embryonic PSM explants. Twenty explants from 10.5-dpc mouse embryos were used for measurement at a single time point. Dashed lines indicate the position of cuts to separate PSM into posterior PSM and anterior PSM. LC-MS/MS, liquid chromatography-tandem mass spectrometry.

(B) Time-course fractional ^{13}C labeling of glycolytic intermediates after culturing of the explants with 0.5 mM U-^{13}C glucose. y Axis in the graphs represents fractional labeling given as a ratio of peak intensities of fully labeled mass isotopomer to the sum of the fully and unlabeled mass isotopomer for a given metabolite. Solid lines indicate posterior PSM while dashed lines indicate anterior PSM. Results are presented as mean ± SD of n = 3 experiments. Student's t test (paired two-tailed distribution) was used to calculate p values. Statistical significance is displayed as *$p < 0.05$ and **$p < 0.01$.

the use of a nutrient-rich medium containing pyruvate, a product of glycolysis, maintains the ability for active TCA cycle and hence energy generation via mitochondrial respiration, the omission of glucose leads to absence of glycolytic flux. As expected, samples cultured in control conditions showed regular PSM development and segmentation during overnight culture, confirmed using both morphological and molecular criteria (Figure 3A). In contrast, we found that culture of PSM explants in pyruvate condition caused severe developmental anomalies (Figure 3B). These were localized mainly in the posterior PSM, while anterior

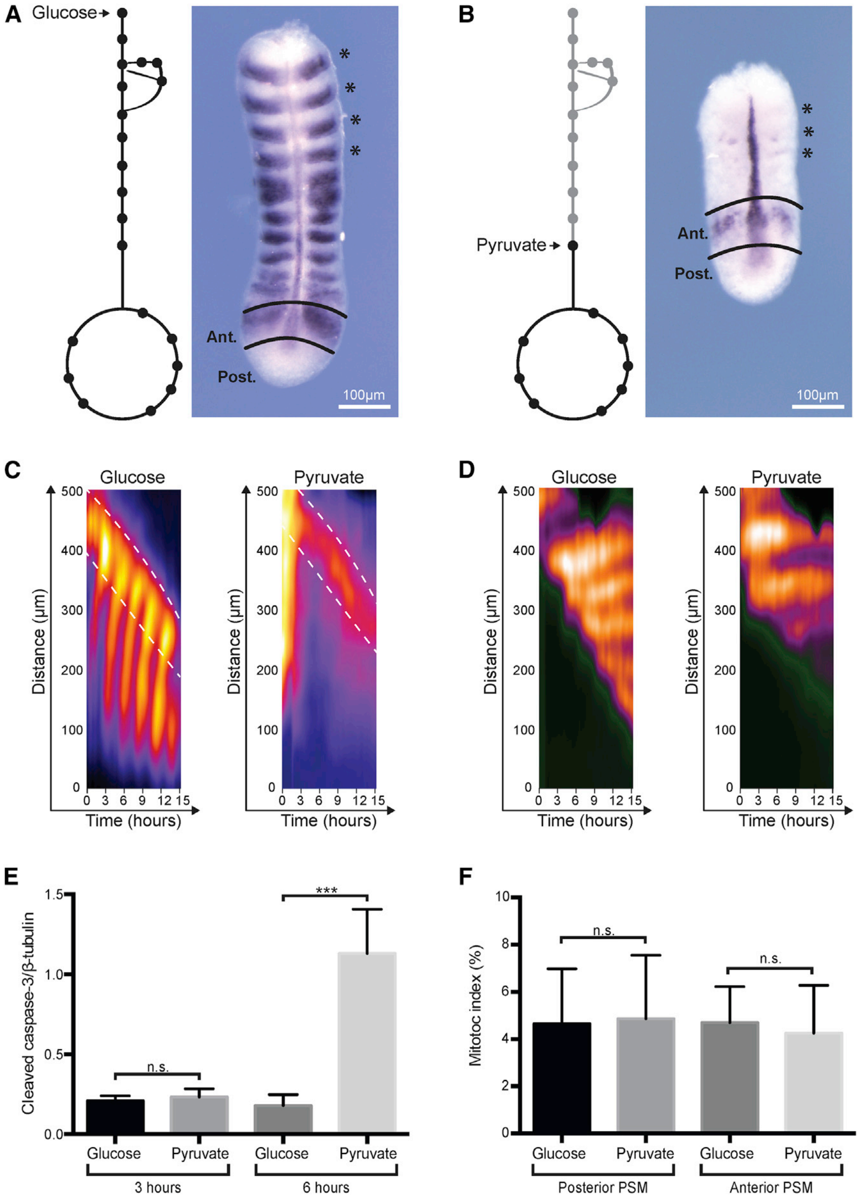

Figure 3. Metabolic Perturbation Causes a Region-Specific PSM Phenotype

(A) Whole-mount mRNA ISH for Lfng, Shh, and Uncx4.1 following 13 hr of culture in control medium (glucose condition). Scheme illustrates entry point of glucose into glycolysis and associated pathways, such as the PPP and TCA cycle, are also shown. Explants cultured with glucose elongate and a wave of cyclic gene Lfng can be detected traversing the PSM from posterior to anterior. Asterisks indicate segments that were present at the onset of the experiment.

(B) Whole-mount mRNA ISH for Lfng, Shh, and Uncx4.1 following 13 hr of culture in pyruvate condition reveals Lfng mRNA expression in the anterior but not the posterior PSM and overall, a truncated PSM. Scheme illustrates entry point of pyruvate as the product of glycolysis. Asterisks indicate segments that were present at the onset of the experiment.

(C) Kymographs depicting activity of LuVeLu, a fluorescent segmentation clock reporter for Lfng (Aulehla et al., 2008) within the PSM (distance from tail bud, posterior, in micrometers). Explants cultured in control medium (0.5 mM glucose in DMEM/F12) displayed regular cyclic activity of LuVeLu and segmentation throughout culture. In samples cultured in pyruvate conditions (10 mM pyruvate in DMEM/F12), dynamic reporter activity is detected in the anterior PSM (highlighted between white dashed lines); however, cyclic activity is not detected in the posterior PSM. n = 8.

(D) Kymographs depicting activity of a Mesp2-GFP reporter line as molecular indication for onset of segmentation. In control conditions (0.5 mM glucose in DMEM/F12), Mesp2-GFP reporter activity is periodically activated in an anterior-to-posterior sequence. Samples cultured in pyruvate conditions (10 mM pyruvate in DMEM/F12) show ongoing molecular segmentation as evidenced by de novo Mesp2 reporter activity in the anterior PSM for several cycles. n = 3.

(E) Quantification of cleaved caspase-3 indicating cells undergoing apoptosis, normalized to β-tubulin levels. Following 3 hr of culture the level of cleaved caspase-3 was not significantly different in explants cultured in control (glucose) compared with pyruvate condition. n = 3. Data are represented as mean ± SD. Statistical significance is displayed as ***$p < 0.001$ and n.s. (not significant).

(F) Analysis of mitotic index of explants cultured for 3 hr in control (glucose) compared with pyruvate condition. The mitotic index was determined using the ratio of phospho-histone H3 immunofluorescence-positive cells to DAPI counterstained cells. Explants cultured in pyruvate do not have a mitotic index significantly different (n.s.) to that of explants cultured in glucose in either the posterior or anterior PSM. n = 3. Data are represented as mean ± SD.

PSM patterning proceeded for several segmentation cycles also in the pyruvate condition. Accordingly, whole-mount mRNA in situ hybridization revealed that after overnight culture in the pyruvate condition the dynamically expressed PSM marker gene and Notch-signaling target Lunatic fringe (Lfng) was lost in the posterior PSM, while expression in the anterior PSM was detected (Figure 3B). Since Lfng is a short-lived, cyclic target of the segmentation clock, we concluded that Lfng continues to be expressed de novo in the anterior PSM, also when culture is performed in pyruvate conditions.

To investigate the temporal onset of this phenotype in more detail, we performed real-time imaging analysis using a Notch-signaling segmentation clock reporter, LuVeLu (Aulehla et al., 2008), as well as a mouse reporter line for Mesp2, a key marker for segment formation (Morimoto et al., 2006). We found that de novo expression of LuVeLu and Mesp2 continues for several segmentation cycles in the anterior PSM in pyruvate culture. In contrast, LuVeLu reporter oscillations were rapidly lost within a few hours in the posterior PSM (Figures 3C and 3D). Prior to the onset of the phenotype we did not detect evidence of significant apoptosis (Figure 3E) or changes at the level of cell-cycle dynamics (Figure 3F). As a control, we cultured samples in a combination of 0.5 mM glucose and 10 mM pyruvate; these samples developed indistinguishably (Figure S3) from samples in the

glucose control condition (Figure 3), indicating that high pyruvate levels in the culture medium do not per se cause any other phenotype.

While the mechanism underlying the rapid deterioration of posterior PSM development remains unclear at this point, especially considering that glucose serves several interconnected metabolic pathways in addition to canonical glycolysis, our combined results do show that anterior and posterior PSM indeed have differential metabolic requirements. Posterior, more glycolytic PSM cells are crucially dependent on the continuous presence of glucose as a carbon source while anterior PSM cells can continue performing molecular segmentation for many hours even in the absence of glucose in the culture medium. Based on these results, we conclude that the metabolic differences we detect within the PSM are indeed mirrored at the functional level.

Development of a Genetically Encoded FRET Sensor for Pyruvate

We next asked how spatial metabolic differences are linked to PSM development and PSM cell differentiation. On addressing this question our goal was to develop a biosensor and mouse reporter line that would enable quantification of pyruvate, a metabolite produced via glycolysis, with spatiotemporal resolution during PSM development. We followed the general strategy of using bacterial proteins that naturally bind metabolites and subsequently undergo a conformational change as the basis to develop fluorescence resonance energy transfer (FRET)-based biosensors (Deuschle et al., 2005). As a pyruvate binding protein we chose *Escherichia coli* pyruvate dehydrogenase regulator (PdhR), in combination with a mTurquoise cp173Venus FRET pair (see STAR Methods for design details). We verified direct pyruvate binding of recombinant mTurquoise-PdhR-cp173Venus in vitro using isothermal titration calorimetry (ITC) (Figure S4A). The quantification of FRET response showed that the acceptor-to-donor emission ratio decreased upon pyruvate binding (Figures 4A and 4B), indicating that this FRET-sensor strategy generated a suitable response. We verified metabolite specificity for pyruvate binding (Figure 4C) and, importantly, verified that the FRET response is not significantly affected by changes of the pH within the physiological range (Figure 4D), with a K_D of 65 μM (Figure S4B).

In vivo experiments using HeLa cells expressing mTurquoise-PdhR-cp173Venus showed a ~20% FRET ratio change upon addition of pyruvate (Figures 4E and S4C), indicating a sufficiently sensitive readout for applications in living cells. While our work was ongoing, two pyruvate biosensors employing a similar strategy using PdhR were reported and successfully used in cell lines, demonstrating the suitability of PdhR as a specific pyruvate binding protein (Peroza et al., 2015; San Martin et al., 2014).

To enable pyruvate quantifications during embryonic development, we generated a pyruvate biosensor mouse line. To this end, we first further optimized the FRET response and used a library approach (Piljic et al., 2011) whereby the PdhR protein was cloned into a variety of donor-acceptor pairs and linker length combinations (Figure S4D). The library screen was performed in HeLa cells and we identified several constructs that showed an enhanced FRET ratio change upon addition of pyruvate, i.e., more than 20% (Figure 4F). The most promising of these improved designs (F41, Figure 4F) was selected for generation of a transgenic mouse reporter line and subsequent in vivo experiments.

Real-Time Imaging Using PYRATES Reporter Line Indicates Dynamic Metabolic Transitions during PSM Differentiation

We next generated a transgenic mouse line expressing pyruvate reporter F41 ubiquitously during embryonic development, and named this mouse line PYRATES (PYRuvATE Sensor). To test whether PYRATES does serve as a pyruvate reporter during mouse embryonic development, we employed a recently developed ex vivo assay that recapitulates PSM patterning and segmentation in primary cell-culture conditions (Figure 5A) (Lauschke et al., 2013). The simplified two-dimensional (2D) geometry of the ex vivo assay greatly facilitates FRET imaging yet maintains the biological context of PSM patterning and differentiation. We first confirmed that addition of pyruvate to the culture medium led to a rapid response at the level of PYRATES FRET ratio in PSM cells cultured as 2D ex vivo assays (Figure 5B).

Next, we tested whether in this context of PSM development PYRATES would also provide, indirectly, an indication for glycolytic activity. To this end, we increased glucose concentration in the culture medium, which we found led to elevated glycolytic activity as evidenced by increased lactate secretion (Figure 5C). We found that culture under an elevated glucose/glycolytic activity condition led to a decrease in PYRATES FRET ratio signal, in turn indicating an increase in steady-state cytosolic pyruvate levels (Figure 5D).

It is critical to point out that in general, steady-state metabolite levels are per se not indicative of underlying metabolic activities. Our data suggest, however, that in this particular context of PSM development the PYRATES biosensor reporter mouse line does mirror changes at the level of glycolytic activity: increased glycolytic activity in the presence of higher glucose concentration does correlate with a decreased PYRATES FRET ratio.

We next used PYRATES to investigate the spatiotemporal dynamics of pyruvate levels during PSM differentiation. We found that after overnight culture of 2D ex vivo assays, which allow cells to transition toward an anterior PSM identity (Lauschke et al., 2013), the PYRATES FRET ratio increased from center to periphery (Figure 5E), indicating spatial differences in steady-state pyruvate levels. Addition of pyruvate to control medium caused, as expected, an immediate decrease in the PYRATES FRET ratio and, importantly, significantly reduced the slope of the PYRATES FRET ratio gradient (Figure S5). Hence, the response to a given amount of excess pyruvate was different along the PYRATES gradient, providing further evidence that the observed spatial differences in PYRATES indeed reflect a gradient of steady-state cytosolic pyruvate levels.

Combined with our previous findings outlined above, this indicated that pyruvate levels (and, indirectly, glycolytic activity) gradually decrease as cells transit toward an anterior PSM-like state. Interestingly, the PYRATES FRET ratio was found to be spatially graded at various glucose concentrations used during sample culture. Hence, while higher glucose levels caused an overall change in FRET signal (mirroring the overall change in pyruvate steady-state levels), the PYRATES FRET ratio was

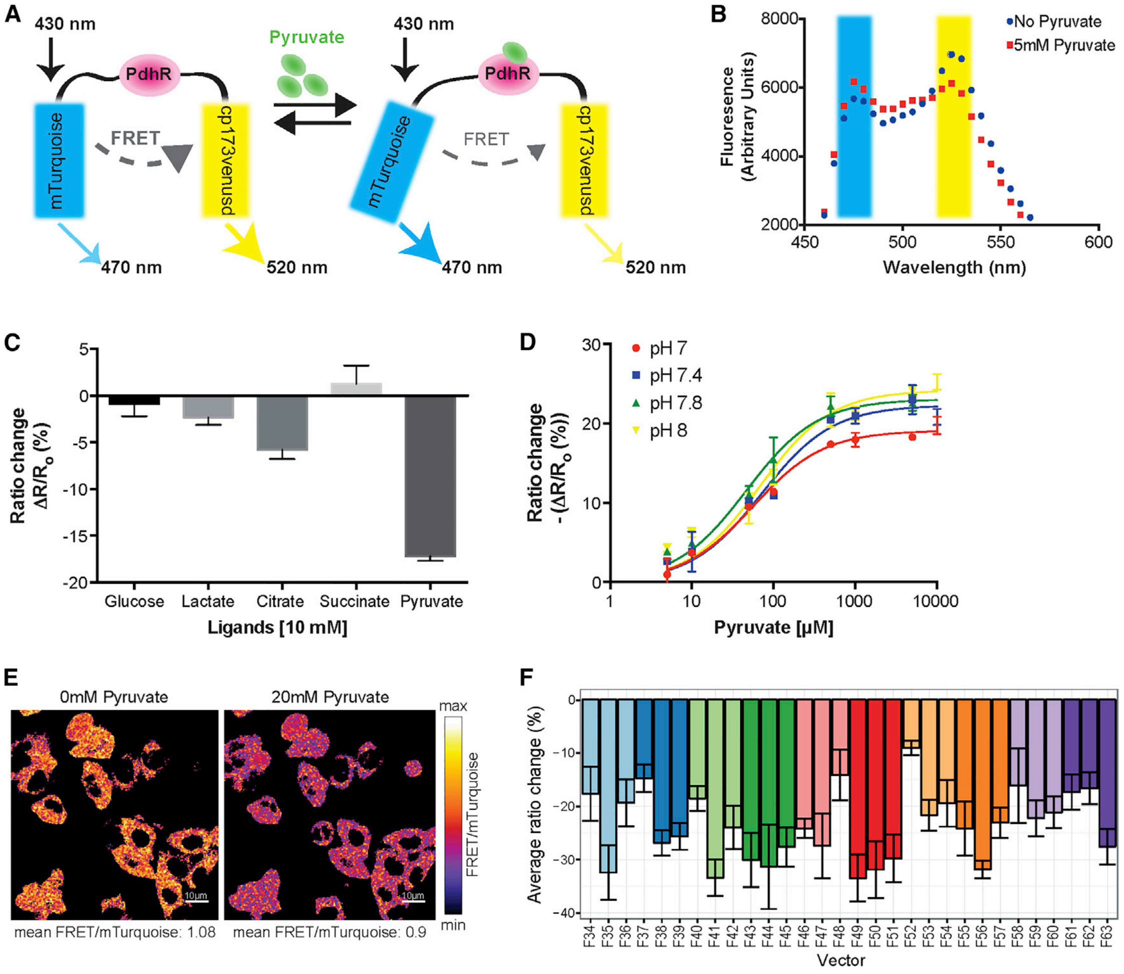

Figure 4. Development of a Genetically Encoded Pyruvate Sensor Based on Fluorescence Resonance Energy Transfer

(A) Design of FRET-based sensor for pyruvate. *E. coli* pyruvate dehydrogenase regulator (PdhR) protein was used as the binding domain. Upon binding pyruvate, the domain undergoes a reversible conformational change, which results in a decrease in FRET from the mTurquoise donor to cp173Venusd acceptor.

(B) Fluorescence emission spectra of recombinant mTurquoise-PdhR-cp173Venusd protein after excitation at 430 nm in the presence (red dotted line) or absence (blue dotted line) of 5 mM pyruvate.

(C) Ligand specificity of the FRET sensor. Purified recombinant mTurquoise-PdhR-cp173-Venusd protein was excited at 430 nm in the presence or absence of different ligands (at 10 mM final concentration). Ratio (R) of FRET acceptor emission intensity to FRET donor emission intensity was calculated before (R_o) and after (R_f) ligand addition and the % ratio change (($R_f - R_o$)/R_o) was obtained and plotted. Data are presented as mean ± SD. n = 3.

(D) Effect of pH on the FRET response of the pyruvate sensor. The effect of pH on the FRET response of recombinant mTurquoise-PdhR-cp173-Venusd protein was studied by incubating the protein for 30 min in the respective pH buffer (pH 7–8) and pyruvate was then titrated in the same buffer. Measurements were recorded and % ratio change ($-(R_f - R_o)/R_o$) was obtained. Data were fitted to one site binding equation using GraphPad Prism software and are presented as mean ± SD. n = 2.

(E) HeLa cells expressing mTurquoise-PdhR-cp173Venusd show drop in FRET ratio (Venus/mTurquoise fluorescence) upon addition of 20 mM pyruvate. Shown are the ratiometric images (Venus/mTurquoise fluorescence) of HeLa cells before and after addition of pyruvate.

(F) Optimization of pyruvate sensor by FRET library screening in HeLa cells. PdhR protein was cloned in between different variants of mTurquoise and Venus attached via 2, 4, or 8 amino acid linker length peptides. The mean ratio change for each construct in HeLa cells (24 cells average for each construct) upon addition of 20 mM pyruvate was quantified from averaged cell traces and plotted. Data are presented as mean ± SD.

nevertheless still spatially graded between central and peripheral PSM cells (Figure 5E). This indirectly indicated that cells are per se not limited in their glycolytic capacity (responding to increase in glucose concentration), and that a dynamic metabolic change, as evidenced by dynamic changes of pyruvate levels, occurs as cells are transitioning toward an anterior PSM identity and, hence, toward differentiation.

To monitor this transition directly, we performed real-time PYRATES imaging during PSM differentiation using the 2D culture assay. We found that at the beginning of the culture, a time point at which all cells still possess a posterior PSM identity (Lauschke et al., 2013), the PYRATES FRET ratio was uniform and not graded (Figures 5F and 5G; Movie S1). However, in peripheral PSM cells that transitioned to a more anterior PSM state during culture, the PYRATES FRET ratio gradually increased, indicating a drop in steady-state pyruvate levels. Interestingly, the de novo formation of the PYRATES FRET ratio gradient occurs in uniform in vitro culture conditions and in a monolayer of

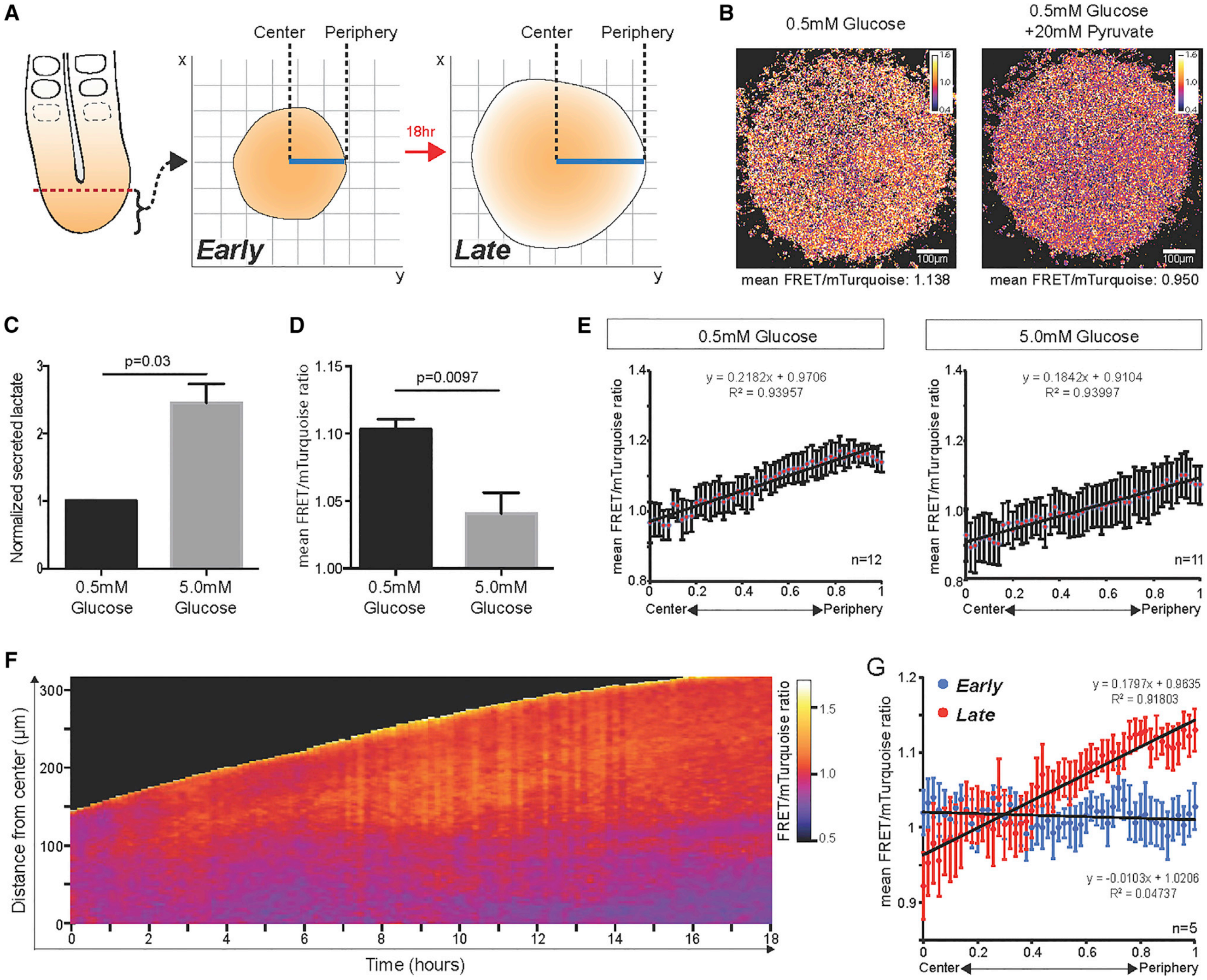

Figure 5. A Pyruvate Gradient Is Formed in the 2D Ex Vivo Segmentation Model of Mouse Embryonic Presomitic Mesoderm

(A) Schematic of the ex vivo culture of mouse embryonic presomitic mesoderm. Posterior PSM was cut (dotted line) and cultured on a fibronectin-coated dish as described previously (Lauschke et al., 2013). Signaling gradients and anterior PSM identity were established after 18 hr of culture. The solid line (blue) was used to measure FRET ratio from center to periphery; this line was rotated 360° to obtain measurements across the whole sample and the average of these measurements was then plotted.

(B) Venus/mTurquoise ratio images of 2D ex vivo cultures (13-hr culture) before and directly after addition of 20 mM pyruvate. Addition of exogenous pyruvate leads to a 16% drop in mean Venus/mTurquoise ratio.

(C) Lactate secretion measured as a proxy for glycolytic activity following overnight ex vivo culture with 0.5 or 5 mM glucose. Lactate secretion at 5 mM glucose was plotted relative to 0.5 mM glucose (n = 13). Data are represented as mean ± SEM. Significantly more lactate is secreted from PSM samples cultured at higher glucose concentration.

(D) Average Venus/mTurquoise values after overnight ex vivo culture with 0.5 or 5 mM glucose (n = 3). Data are represented as mean ± SEM. Significantly lower FRET is measured in PSM samples cultured at a higher glucose concentration, indicating higher cytoplasmic pyruvate levels.

(E) Ex vivo culture of mouse PSM expressing PYRATES shows a gradient of FRET ratio from center to periphery at 0.5 mM glucose (n = 12) and at a higher glucose concentration of 5 mM (n = 11). Mean Venus/mTurquoise values for each relative spatial coordinate was obtained and plotted; the center coordinate was set to 0 and the periphery set to 1. Data are presented as mean ± SEM.

(F) Kymograph showing real-time quantification of PYRATES FRET ratio (Venus/mTurquoise fluorescence) during 2D ex vivo culture with 0.5 mM glucose for 18 hr. Kymograph of the FRET ratio from center to periphery (y axis) is plotted at 10-min intervals (x axis).

(G) Quantification of PYRATES FRET ratio gradients in early and late 2D ex vivo PSM cultures with 0.5 mM glucose. Mean Venus/mTurquoise fluorescence values for each relative spatial coordinate were obtained and plotted after early (0 hr) and late (13 hr) ex vivo culture (n = 5). Data are presented as mean ± SEM; linear trendline and fit are indicated.

cells. Accordingly, access to nutrients, such as glucose or oxygen, is not likely to generate any bias within the in vitro culture, which otherwise could explain the observed differences in the metabolic state. Rather, these findings support the view that the change in metabolic activity along the PSM is indeed intrinsically linked to PSM cell differentiation.

DISCUSSION

Aerobic Glycolysis in Organogenesis-Stage Mouse Embryos

In this work, we analyzed the functional metabolic state of organogenesis-stage mouse embryos with spatial and temporal resolution. Focusing on mouse embryo PSM development, we reveal spatial metabolic differences, with higher levels of glucose carrier, glycolytic enzyme expression, and glycolytic flux found in posterior compared with anterior PSM cells. Concomitantly, our analysis indicated that oxygen consumption is higher in anterior PSM cells than in posterior counterparts. The ability of posterior PSM cells to show a metabolic signature of high glycolytic activity relative to the anterior PSM even in the presence of oxygen, i.e., aerobic glycolysis, is a phenotype that appears reminiscent of the well-known Warburg effect (Koppenol et al., 2011; Warburg et al., 1927). The functional role of the Warburg effect has been extensively discussed, especially in the cancer biology field (Koppenol et al., 2011; Senyilmaz and Teleman, 2015; Vander Heiden et al., 2009; Ward and Thompson, 2012; Zu and Guppy, 2004). High glycolytic flux is commonly proposed to serve increased biosynthetic and anabolic demands of a cell, essentially reflecting the high proliferative potential, for instance, of cancer cells (Lunt and Vander Heiden, 2011; Vander Heiden et al., 2009). In contrast to the Warburg state in cancer cells, we find that increased aerobic glycolysis in posterior, undifferentiated PSM cells does not seem to reflect differences at the level of proliferative activity, as we do not find spatial differences in proliferation within the PSM. We do show, however, that these metabolic differences are reflected at the functional level, as we reveal that anterior and posterior PSM cells have different requirements for the presence of glucose as carbon source. The functional role of a glycolytic activity gradient during the process of PSM development remains to be addressed in future studies.

A Pyruvate Biosensor Mouse Line Enables Real-Time Metabolite Measurements within the PSM

We generated a pyruvate FRET-sensor reporter mouse line that enables the quantification of cytoplasmic pyruvate levels with spatiotemporal resolution. Biosensors have received increasing attention due to their potential to monitor small molecules and metabolites within living cells; accordingly, the repertoire of available sensors is steadily increasing and improving (Hou et al., 2011). As pointed out above, while we were in the process of generating the biosensor mouse reporter line other groups reported the generation of pyruvate FRET sensors and successful application in cell cultures (Peroza et al., 2015; San Martin et al., 2014). Overall, the performance of our pyruvate FRET sensor in a cell-culture model is very comparable with these previously developed pyruvate sensors. In addition, these studies extensively validated the specificity of PdhR as a pyruvate binding protein (Peroza et al., 2015; San Martin et al., 2014). Of note, while it was shown that citrate at low-millimolar concentration elicited a small response of the pyruvate sensor (San Martin et al., 2014), as we also find in our study (Figure 4C), this previous study confirmed that a response was not observed when citrate was used at physiological (i.e., low micromolar) concentrations (San Martin et al., 2014). As also seen in our library screen approach, it is known that already very minor differences in FRET-reporter design, such as linker lengths, can have pronounced impact on reporter performance. It will therefore be important to compare the performance of these different pyruvate sensor designs directly in vivo and within the same application. We generated a pyruvate FRET-sensor reporter mouse line and validated its suitability to function as a pyruvate sensor during embryonic development. In addition, we provide evidence that in this context of mouse PSM development, it indirectly mirrors glycolytic activity. Future work will need to determine whether this correlation is specific for the context of PSM. More generally, the PYRATES biosensor mouse reporter line might prove to be a valuable resource enabling pyruvate measurements with spatiotemporal resolution in a wide range of different applications in developmental and cell biology.

Evidence for a Glycolytic Activity Gradient Intrinsically Linked to PSM Development

We have provided evidence that a gradient of glycolytic activity forms during PSM development, both in vivo and also under uniform in vitro conditions, giving rise to the question of what is the underlying control mechanism. In vivo, local environmental conditions, such as limited access to nutrients or oxygen, could impose altered metabolic activity in specific PSM regions. Such a mechanism has indeed been found to (at least partially) explain elevated glycolysis and metabolic rewiring in hypoxic tumor environments (Moreno-Sánchez et al., 2007; Tennant et al., 2009; Zu and Guppy, 2004) and hypoxic stem cell niches (Pattappa et al., 2011; Shyh-Chang et al., 2013; Suda et al., 2011). In contrast, we provide evidence that the spatial metabolic differences we observe are not due to limited nutritional supply. First, our analysis is done in organogenesis-stage mouse embryos, a developmental time at which placental function is fully established and access to oxygen and nutrients such as glucose is not expected to differ regionally. Second, we find that after long-term (21 hr) culture of PSM explants in uniform in vitro conditions, glucose uptake remains high within posterior compared with anterior PSM cells, indicating that elevated glycolytic activity in the posterior PSM is an intrinsic property (Figure S1). Most importantly, we provide evidence that even in primary 2D cell-culture conditions, cells exhibit spatially non-uniform pyruvate levels, indicating the de novo formation of a glycolytic activity gradient in vitro. We find that while in vitro cultured cells are not restricted in their glycolytic capacity and respond to increasing levels of substrate, i.e., glucose (Figure 5E), the glycolytic activity gradient still forms as cells with anterior PSM identity show relatively lower glycolytic activity than cells with posterior PSM identity.

Our combined findings hence indicate that glycolytic activity in PSM cells is dynamically regulated at the level of glucose uptake carrier and glycolytic enzyme expression, possibly by signals that also control PSM differentiation. Several of these signaling pathways, such as the Fgf, Wnt, and retinoic acid signaling pathways, display graded activities along the PSM and have been shown to control PSM cell differentiation (Aulehla and Pourquié, 2010). These pathways have been implicated in several studies as affecting the metabolic state (Saumet et al., 2012; Sethi and Vidal Puig, 2010). For instance, Wnt signaling has been shown to control cell metabolism in normal and cancer cells, by virtue of regulating expression but also by protein stability of metabolic

enzymes, in a context-dependent manner (Kim et al., 2009; Pate et al., 2014; Sethi and Vidal Puig, 2010; Sherwood, 2015).

Interestingly, a companion study (Oginuma et al., 2017 [this issue of *Developmental Cell*]) indeed revealed first evidence for a functional interplay between Fgf/Wnt signaling with a glycolytic gradient in the context of PSM segmentation in chick embryos. The ability to reveal metabolite levels and potentially metabolic transitions with spatiotemporal resolution will be critical in future studies that will address how metabolic and signaling gradients are mechanistically and functionally linked within the context of embryonic development.

STAR★METHODS

Detailed methods are provided in the online version of this paper and include the following:

- KEY RESOURCES TABLE
- CONTACT FOR REAGENT AND RESOURCE SHARING
- EXPERIMENTAL MODEL AND SUBJECT DETAILS
  - Transgenesis, Mouse Strains and Animal Work
- METHOD DETAILS
  - Mouse PSM Explant and 2D PSM Ex Vivo Cultures
  - Mouse PSM Whole Mount In Situ Hybridization Screen
  - Immunoblot Analysis
  - Whole Mount Immunohistochemistry, Optical Clearing and Optical Sectioning
  - Lactate Measurement Assay
  - Basal Oxygen Consumption Rate Measurements
  - 2-NBDG Uptake and Imaging
  - Glycolytic Labeling Kinetics from ^{13}C-Isotope-Tracing
  - DNA Cloning
  - Expression and Purification of mTurquoise-PdhR-cp173 Venus Protein
  - In Vitro FRET Measurements
  - Isothermal Titration Calorimetry
  - Cell Culture and Transfection
  - Two-Photon Microscopy
  - FRET Imaging and Image Processing
- QUANTIFICATION AND STATISTICAL ANALYSIS

SUPPLEMENTAL INFORMATION

Supplemental Information includes five figures, one table, and one movie and can be found with this article online at http://dx.doi.org/10.1016/j.devcel.2017.01.015.

AUTHOR CONTRIBUTIONS

Conceptualization, A.A., U.S., and C.S.; Investigation, V.B., N.P., M.T.S., A.K., K.F.S., J.K., F.S., and A.A.; Writing – Original Draft, V.B., N.P., M.T.S., and A.A.; Writing – Review & Editing, V.B., N.P., M.T.S., A.A., U.S., and C.S.; Supervision, A.A., U.S., and C.S.

ACKNOWLEDGMENTS

We thank Ivica Lehotska and Thiebault Lequeu for their excellent experimental help with PYRATES real-time imaging and analysis and Hidenobu Miyazawa for help with the revision of the manuscript. We thank the EMBL Transgenic Service/Yvonne Petersen for generation of the PYRATES transgenic mouse line. We thank Vladimir Rybin, Protein Core Facility, EMBL, for ITC analysis. This work was supported by EMBL Advanced Light Microscopy, Protein Core Facility, and Laboratory Animal Resource facilities. V.B. was supported by the "EMBL Interdisciplinary Postdoc Program" under the Marie Curie COFUND Actions (grant 18877 [Cofund 2]). This work was supported by the Cluster of Excellence Cellular Networks – CellNetworks grant (EcTOP6, Metabolism and Development), University of Heidelberg.

Received: June 5, 2016
Revised: December 1, 2016
Accepted: January 27, 2017
Published: February 27, 2017

REFERENCES

Agathocleous, M., and Harris, W.A. (2013). Metabolism in physiological cell proliferation and differentiation. Trends Cell Biol. *23*, 484–492.

Aulehla, A., and Pourquié, O. (2010). Signaling gradients during paraxial mesoderm development. Cold Spring Harb. Perspect. Biol. *2*, a000869.

Aulehla, A., Wehrle, C., Brand-Saberi, B., Kemler, R., Gossler, A., Kanzler, B., and Herrmann, B.G. (2003). Wnt3a plays a major role in the segmentation clock controlling somitogenesis. Dev. Cell *4*, 395–406.

Aulehla, A., Wiegraebe, W., Baubet, V., Wahl, M.B., Deng, C., Taketo, M., Lewandoski, M., and Pourquié, O. (2008). A beta-catenin gradient links the clock and wavefront systems in mouse embryo segmentation. Nat. Cell Biol. *10*, 186–193.

Bajard, L., Morelli, L.G., Ares, S., Pecreaux, J., Julicher, F., and Oates, A.C. (2014). Wnt-regulated dynamics of positional information in zebrafish somitogenesis. Development *141*, 1381–1391.

Buescher, J.M., Moco, S., Sauer, U., and Zamboni, N. (2010). Ultrahigh performance liquid chromatography-tandem mass spectrometry method for fast and robust quantification of anionic and aromatic metabolites. Anal. Chem. *82*, 4403–4412.

Cambray, N., and Wilson, V. (2002). Axial progenitors with extensive potency are localised to the mouse chordoneural hinge. Development *129*, 4855–4866.

Cankaya, M., Hernandez, A., Ciftci, M., Beydemir, S., Ozdemir, H., Budak, H., Gulcin, I., Comakli, V., Emircupani, T., Ekinci, D., et al. (2007). An analysis of expression patterns of genes encoding proteins with catalytic activities. BMC Genomics *8*, 232.

Deuschle, K., Fehr, M., Hilpert, M., Lager, I., Lalonde, S., Looger, L.L., Okumoto, S., Persson, J., Schmidt, A., and Frommer, W.B. (2005). Genetically encoded sensors for metabolites. Cytometry A *64*, 3–9.

Diez del Corral, R., Olivera-Martinez, I., Goriely, A., Gale, E., Maden, M., and Storey, K. (2003). Opposing FGF and retinoid pathways control ventral neural pattern, neuronal differentiation, and segmentation during body axis extension. Neuron *40*, 65–79.

Dubrulle, J., McGrew, M.J., and Pourquie, O. (2001). FGF signaling controls somite boundary position and regulates segmentation clock control of spatiotemporal Hox gene activation. Cell *106*, 219–232.

Hama, H., Kurokawa, H., Kawano, H., Ando, R., Shimogori, T., Noda, H., Fukami, K., Sakaue-Sawano, A., and Miyawaki, A. (2011). Scale: a chemical approach for fluorescence imaging and reconstruction of transparent mouse brain. Nat. Neurosci. *14*, 1481–1488.

Hou, B.H., Takanaga, H., Grossmann, G., Chen, L.Q., Qu, X.Q., Jones, A.M., Lalonde, S., Schweissgut, O., Wiechert, W., and Frommer, W.B. (2011). Optical sensors for monitoring dynamic changes of intracellular metabolite levels in mammalian cells. Nat. Protoc. *6*, 1818–1833.

Hubaud, A., and Pourquie, O. (2014). Signalling dynamics in vertebrate segmentation. Nat. Rev. Mol. Cell Biol. *15*, 709–721.

Kim, N.G., Xu, C., and Gumbiner, B.M. (2009). Identification of targets of the Wnt pathway destruction complex in addition to beta-catenin. Proc. Natl. Acad. Sci. USA *106*, 5165–5170.

Koppenol, W.H., Bounds, P.L., and Dang, C.V. (2011). Otto Warburg's contributions to current concepts of cancer metabolism. Nat. Rev. Cancer *11*, 325–337.

Krol, A.J., Roellig, D., Dequeant, M.-L., Tassy, O., Glynn, E., Hattem, G., Mushegian, A., Oates, A.C., and Pourquie, O. (2011). Evolutionary plasticity of segmentation clock networks. Development *138*, 2783–2792.

Lauschke, V.M., Tsiairis, C.D., Francois, P., and Aulehla, A. (2013). Scaling of embryonic patterning based on phase-gradient encoding. Nature *493*, 101–105.

Lunt, S.Y., and Vander Heiden, M.G. (2011). Aerobic glycolysis: meeting the metabolic requirements of cell proliferation. Annu. Rev. Cell Dev. Biol. *27*, 441–464.

Moreno, T.A., and Kintner, C. (2004). Regulation of segmental patterning by retinoic acid signaling during *Xenopus* somitogenesis. Dev. Cell *6*, 205–218.

Moreno-Sánchez, R., Rodríguez-Enríquez, S., Marín-Hernández, A., and Saavedra, E. (2007). Energy metabolism in tumor cells. FEBS J. *274*, 1393–1418.

Morimoto, M., Kiso, M., Sasaki, N., and Saga, Y. (2006). Cooperative Mesp activity is required for normal somitogenesis along the anterior-posterior axis. Dev. Biol. *300*, 687–698.

Niwa, H., Yamamura, K., and Miyazaki, J. (1991). Efficient selection for high-expression transfectants with a novel eukaryotic vector. Gene *108*, 193–199.

Oginuma, M., Moncuquet, P., Xiong, F., Karoly, E., Chal, J., Guevorkian, K., and Pourquié, O. (2017). A gradient of glycolytic activity coordinates FGF and Wnt signaling during elongation of the body axis in amniote embryos. Dev. Cell *40*, this issue, 342–353.

Ozbudak, E.M., Tassy, O., and Pourquie, O. (2010). Spatiotemporal compartmentalization of key physiological processes during muscle precursor differentiation. Proc. Natl. Acad. Sci. USA *107*, 4224–4229.

Pate, K.T., Stringari, C., Sprowl-Tanio, S., Wang, K., TeSlaa, T., Hoverter, N.P., McQuade, M.M., Garner, C., Digman, M.A., Teitell, M.A., et al. (2014). Wnt signaling directs a metabolic program of glycolysis and angiogenesis in colon cancer. EMBO J. *33*, 1454–1473.

Pattappa, G., Heywood, H.K., de Bruijn, J.D., and Lee, D.A. (2011). The metabolism of human mesenchymal stem cells during proliferation and differentiation. J. Cell Physiol. *226*, 2562–2570.

Peroza, E.A., Boumezbeur, A.H., and Zamboni, N. (2015). Rapid, randomized development of genetically encoded FRET sensors for small molecules. Analyst *140*, 4540–4548.

Piljic, A., de Diego, I., Wilmanns, M., and Schultz, C. (2011). Rapid development of genetically encoded FRET reporters. ACS Chem. Biol. *6*, 685–691.

Rühl, M., Le Coq, D., Aymerich, S., and Sauer, U. (2012). ^{13}C-flux analysis reveals NADPH-balancing transhydrogenation cycles in stationary phase of nitrogen-starving Bacillus subtilis. J. Biol. Chem. *287*, 27959–27970.

San Martin, A., Ceballo, S., Baeza-Lehnert, F., Lerchundi, R., Valdebenito, R., Contreras-Baeza, Y., Alegria, K., and Barros, L.F. (2014). Imaging mitochondrial flux in single cells with a FRET sensor for pyruvate. PLoS One *9*, e85780.

Saumet, A., Vetter, G., Bouttier, M., Antoine, E., Roubert, C., Orsetti, B., Theillet, C., and Lecellier, C.H. (2012). Estrogen and retinoic acid antagonistically regulate several microRNA genes to control aerobic glycolysis in breast cancer cells. Mol. Biosyst. *8*, 3242–3253.

Sawada, A., Shinya, M., Jiang, Y.J., Kawakami, A., Kuroiwa, A., and Takeda, H. (2001). Fgf/MAPK signalling is a crucial positional cue in somite boundary formation. Development *128*, 4873–4880.

Schindelin, J., Arganda-Carreras, I., Frise, E., Kaynig, V., Longair, M., Pietzsch, T., Preibisch, S., Rueden, C., Saalfeld, S., Schmid, B., et al. (2012). Fiji: an open-source platform for biological-image analysis. Nat. Methods *9*, 676–682.

Senyilmaz, D., and Teleman, A.A. (2015). Chicken or the egg: Warburg effect and mitochondrial dysfunction. F1000Prime Rep. *7*, 41.

Sethi, J.K., and Vidal Puig, A. (2010). Wnt signalling and the control of cellular metabolism. Biochem. J. *427*, 1–17.

Shepard, T.H., Tanimura, T., and Park, H.W. (1997). Glucose absorption and utilization by rat embryos. Int. J. Dev. Biol. *41*, 307–314.

Sherwood, V. (2015). WNT signaling: an emerging mediator of cancer cell metabolism? Mol. Cell Biol. *35*, 2–10.

Shyh-Chang, N., Daley, G.Q., and Cantley, L.C. (2013). Stem cell metabolism in tissue development and aging. Development *140*, 2535–2547.

Stein, F., Kress, M., Reither, S., Piljic, A., and Schultz, C. (2013). FluoQ: a tool for rapid analysis of multiparameter fluorescence imaging data applied to oscillatory events. ACS Chem. Biol. *8*, 1862–1868.

Suda, T., Takubo, K., and Semenza, G.L. (2011). Metabolic regulation of hematopoietic stem cells in the hypoxic niche. Cell Stem Cell *9*, 298–310.

Tennant, D.A., Duran, R.V., Boulahbel, H., and Gottlieb, E. (2009). Metabolic transformation in cancer. Carcinogenesis *30*, 1269–1280.

Vander Heiden, M.G., Cantley, L.C., and Thompson, C.B. (2009). Understanding the Warburg effect: the metabolic requirements of cell proliferation. Science *324*, 1029–1033.

Venters, S.J., Hultner, M.L., and Ordahl, C.P. (2008). Somite cell cycle analysis using somite-staging to measure intrinsic developmental time. Dev. Dyn. *237*, 377–392.

Warburg, O., Wind, F., and Negelein, E. (1927). The metabolism of tumors in the body. J. Gen. Physiol. *8*, 519–530.

Ward, P.S., and Thompson, C.B. (2012). Metabolic reprogramming: a cancer hallmark even Warburg did not anticipate. Cancer Cell *21*, 297–308.

Watson, E.D., and Cross, J.C. (2005). Development of structures and transport functions in the mouse placenta. Physiology (Bethesda) *20*, 180–193.

Yuan, J., Bennett, B.D., and Rabinowitz, J.D. (2008). Kinetic flux profiling for quantitation of cellular metabolic fluxes. Nat. Protoc. *3*, 1328–1340.

Zu, X.L., and Guppy, M. (2004). Cancer metabolism: facts, fantasy, and fiction. Biochem. Biophy. Res. Commun. *313*, 459–465.

STAR★METHODS

KEY RESOURCES TABLE

Reagent or Resource	Source	Identifier
Antibodies		
Aldolase A	Proteintech	Cat#11217-1-AP; RRID: AB_2224626
Triose phosphate isomerase	Acris	Cat#AP16324PU-N; RRID: AB_1928285
Gapdh	Millipore	Cat#MAB374; RRID: AB_2107445
Pkm 1/2	Cell Signaling	Cat#3190; RRID: AB_2163695
Beta tubulin	Millipore	Cat#05-661; RRID: AB_309885
Cleaved caspase 3 (Asp175)	Cell Signaling	Cat#9661; RRID: AB_2341188
Phospho-Histone H3 (Ser10)(D2C8)	Cell Signaling	Cat#3642; RRID: AB_1549590
Histone H2B	Millipore	Cat#07-371; RRID: AB_310561
Chemicals, Peptides, and Recombinant Proteins		
2-NBDG fluorescent glucose analog	Thermo Fisher	Cat#N13195
DMEM/F12 without Glucose, Pyruvate, Phenol Red	Cell Culture Technologies	N/A
D-Glucose (U-13C6, 99%)	Cambridge Isotope Laboratories	Cat#CLM-1396-2
Critical Commercial Assays		
Lactate Assay kit	Biovision	Cat#K607
Deposited Data		
Kyoto Encyclopedia of Genes and Genomes	KEGG	http://www.genome.jp/kegg/
Experimental Models: Organisms/Strains		
Mouse: LuVeLu:CD1-Tg(Lfng-YFP/PEST)OP	Aulehla et al., 2008	N/A
Mouse: MESP2-GFP: ICR.Cg-Mesp2$^{tm(GFP)}$/YsaRbrc	Morimoto et al., 2006	Bioresources of Riken BRC RBRC:01862; RRID:IMSR_RBRC01862
Mouse: PYRATES:CD1-Tg(CAGGS-mTurquoise/PdhR/cp173Venus)AAu	This paper	N/A
Recombinant DNA		
pECFP-C1 vector	Piljic et al., 2011	N/A
Software and Algorithms		
Fiji	Schindelin et al., 2012	https://fiji.sc/
FluoQ macro	Stein et al., 2013	https://github.com/fstein/FluoQ
Fast 2D peak finder for Matlab	Mathworks Adi Nathan (Stanford University)	https://de.mathworks.com/matlabcentral/fileexchange/37388-fast-2d-peak-finder?s_tid=srchtitle
Other		
LSM 780 laser-scanning microscope (Objectives Plan-Apochromat 10x/0.45, 20x/0.8)	Zeiss	N/A
SP2-AOBS confocal microscope (oil immersion objective 40x/1.4)	Leica microsystems	N/A

CONTACT FOR REAGENT AND RESOURCE SHARING

Please direct all methodological and resource sharing questions to Alexander Aulehla (aulehla@embl.de).

EXPERIMENTAL MODEL AND SUBJECT DETAILS

Transgenesis, Mouse Strains and Animal Work

The *PYRATES* line was generated *via* pronuclear injection of pCKI-PYRATES (see Method Details section), *LuVeLu* and *Mesp2-GFP* lines were described previously (Aulehla et al., 2008; Morimoto et al., 2006). The *Mesp2-GFP* line was obtained from RIKEN BRC

through the National Bio-Resource Project of the MEXT, Japan. All animals are housed in the EMBL animal facilities under veterinarian supervision and are treated following the guidelines of the European Commission, revised directive 2010/63/EU and AVMA guidelines 2007.

METHOD DETAILS

Mouse PSM Explant and 2D PSM Ex Vivo Cultures

Culture experiments were done as described previously(Lauschke et al., 2013). Mouse embryos were collected at 10.5-dpc in PBS with 1% BSA (Equitech-Bio) and 0.5mM glucose. PSM explants consisted of the entire PSM plus three pre-formed somites. Samples for the 2D PSM ex vivo cultures were prepared as described (Lauschke et al., 2013). The culture medium was DMEM/F12 (without glucose, pyruvate and phenol red, Cell culture technologies), supplemented with 0.5mM Glucose and 1% BSA, if not specified otherwise. Additional metabolites were added in the concentrations indicated in the experiments. Explants were cultured in pre-equilibrated culture medium in 8-well chamber slides (Lab-Tek #155411) in a humidified chamber at 37°C, 5% CO_2 and 60% O_2 (PSM explant cultures) and 37°C, 5% CO_2 in ambient air (2D PSM ex vivo cultures).

Mouse PSM Whole Mount In Situ Hybridization Screen

Whole mount in situ hybridization (WISH) with Digoxigenin-labeled RNA probes was performed as described previously (Aulehla et al., 2008). Probes used in the screen were generated as follows; candidate genes from key metabolic pathways were identified with KEGG (Kyoto Encyclopedia of Genes and Genomes). Primers (IDT) were designed for chosen genes using BatchPrimer3 v1.0 software to amplify ~800bp of 3'UTR, using genomic DNA. The T7 promoter sequence (CAGAGATGCATAATACGACTCACTATAGGGAGA) was added to the 5' end of reverse primers to enable in vitro transcription of the PCR products. After WISH and color reaction, embryos were imaged on 1% agar/PBS plates with a MZ16F stereomicroscope (Leica) using a DFC420C Digital camera (Leica) and Leica Application Suite 3.8.0 Software. Basic linear contrast adjustments were applied to images. Scale bars were attributed to images using a calibration slide (Rapp OptoElectronic).

Immunoblot Analysis

Cell and tissue lysates were made in Laemmli with 5% RIPA buffer, denatured by heating at 95°C for 5 minutes, separated by SDS-PAGE under reducing conditions, transferred to an Immobilon-P nitrocellulose membrane (Millipore) and analyzed by immunoblotting. Primary antibodies (with dilutions) used were; Anti-AldolaseA (Proteintech, 1/5000), Anti-Tpi (Acris, 1/5000), Anti-Gapdh (Millipore, 1/5000), Anti-Pkm1/2 (Cell signaling, 1/5000), Anti-H2B (Millipore, 1/10000), Anti-β-tubulin (Millipore, 1/10000) and Anti-Cleaved Caspase C3 (Cell signaling, 1/1000). Chemiluminescence was detected on Amersham Hyperfilms (GE Healthcare) according to the manufacturer's instructions. Densitometric quantifications of bands were performed with NIH Image J software.

Whole Mount Immunohistochemistry, Optical Clearing and Optical Sectioning

Explants were fixed after the specified culture period for 30 minutes in 4% formaldehyde and processed for imunnohistochemistry. Primary antibody: phospho-Histone H3 (D2C8) Antibody (Cell Signaling, 1/1000). Secondary antibodies: goat-anti-rabbit Alexa-488 (Invitrogen) or goat-anti-rabbit Alexa-568 (Invitrogen). Nuclei were then counterstained with DAPI (InnoGenex CS-2010–06). For imaging samples were optically cleared via incubation with Sca*l*e reagents (Hama et al., 2011). In brief, samples were first incubated in Sca*l*e B4 solution (8 M Urea, 0.1% Triton X-100) at 4°C for 2 days and then transferred into Scale U2 solution (4 M Urea, 30% glycerol, 0.1% Triton X-100) for a minimum of 4 weeks. Samples were imaged on a LSM780 laser-scanning microscope (Zeiss) through a 10x Plan- Apochromat objective, optical sections acquired at 3μm intervals. DAPI was excited using a 405 nm laser diode, Alexa-568 using a 561-nm laser diode and Alexa-488 using a two-photon laser at 960 nm.

For determination of mitotic indices, images were pre-processed in Fiji by Gaussian blur filtering. Anti-Phospho-H3- or DAPI-stained nuclei were detected in each optical section by determining local maxima using Matlab. The script was based on the published Matlab function FastPeakFind by Adi Natan (Stanford University). Mitotic index was determined as number of Phospho-H3-positive nuclei relative to DAPI-positive nuclei in the indicated regions averaged over all optical sections.

Lactate Measurement Assay

Fluorometric lactate measurements were performed with the Lactate Assay Kit (Biovision) according to manufacturers instructions, the reaction volume was reduced to 50μl.

Basal Oxygen Consumption Rate Measurements

Embryonic explants were dissected from 10.5-dpc mouse embryos and were incubated in DMEM/F12 (without glucose, pyruvate and phenol red (Cell culture technologies), supplemented with 0.5mM glucose and 1% BSA (Equitech-Bio) at 37°C, 5% CO_2, 60% O_2 for 30 min. Explants were then dissected into posterior and anterior PSM fragments in equilibrated medium and were then transferred to a calibrated Clarke type oxygen electrode (Hansatech Instruments) maintained at 37°C. Basal oxygen consumption rate was measured for posterior and anterior PSM fragments (n=20 per reading) and then normalized to the total cell number.

2-NBDG Uptake and Imaging

2-NBDG ((2-(*N*-(7-Nitrobenz-2-oxa-1,3-diazol-4-yl)Amino)-2-Deoxyglucose) (Life Technologies) stock solution was made in DMSO at a concentration of 200mM, aliquoted and stored at -20°C. To measure 2-NBDG uptake in embryonic explants, the culture medium was removed and the explants were washed three times with equilibrated culture medium without glucose and were then incubated in culture medium containing 2-NBDG at a final concentration of 0.5mM for 30 minutes at 37°C, 5% CO_2, 60% O_2. After incubation, explants were washed with culture medium and then imaged on a LSM780 laser-scanning microscope (Zeiss) using two-photon excitation. Samples were excited with 900nm laser and fluorescence emission recorded from 520 to 560nm. Images were processed in Fiji (Schindelin et al., 2012), intensities in the anterior and posterior PSM were measured following a 10μm Gaussian Blur.

Glycolytic Labeling Kinetics from ^{13}C-Isotope-Tracing

To measure dynamics of glucose metabolism, explants were collected from CD1 mouse embryos at 10.5-dpc with five pre-formed somites and cultured in DMEM/F12 (without glucose, pyruvate and phenol red (Cell culture technologies), supplemented with 0.5mM glucose and 1% BSA (Equitech-Bio) at 37°C, 5% CO_2, 60% for 30 minutes. Explants were then dissected in culture medium into anterior and posterior PSM fragments, these were then placed in separate net wells. Twenty explants were used for a single time point measurement. Before isotope switch of naturally labeled glucose to uniformly labeled U-^{13}C- glucose, the explants were further incubated for 10 minutes in culture medium containing 0.5mM naturally labeled glucose to prevent effects due to sudden media change as described (Yuan et al., 2008). Subsequently, net wells with the different PSM regions were rapidly transferred to culture medium with 0.5mM U-^{13}C- glucose and incubated for the indicated time. Following culture, the samples were washed three times in 75mM ammonium carbonate (pH 7.4) and quenched by snap freezing in liquid nitrogen. Metabolites were extracted using hot ethanol, preheated at 75°C. To this end, after each ethanol addition the sample was placed at 75°C whilst shaking at 1400rpm for 90 seconds and then centrifuged for 30 seconds. The samples were kept on ice before concentration in a speedvac at 0.12 mbar and 1500 rpm (Christ, Germany) at room temperature until the samples were completely dry (~4 hours). Samples were then resuspended in H_2O and analyzed with ultra-high-pressure chromatography-coupled tandem mass spectrometry as described before (Buescher et al., 2010; Rühl et al., 2012). Due to the low biomass, the measurements were close to the detection limit. Therefore, only the unlabeled and fully labeled mass isotopomers of a metabolite were considered. The mass isotopomers were normalized to the total metabolite pool (i.e. unlabeled + fully labeled).

DNA Cloning

PdhR ORF was PCR amplified from *E. coli* genomic DNA with primers containing *Age*I, *Mlu*I sites. The amplified product was cloned in TOPO vector (Life technologies) and sequence verified. The plasmid was digested with *Age*I and *Mlu*I and the insert was subcloned in pECFP-C1 modified vector, F40 (Piljic et al., 2011) to get mTurquoise at the N-terminus and cp173 Venus at the C-terminus of PdhR. The whole gene cassette was then excised with *Nhe*I and *BamH*I and then digested with *Nco*I, which was subsequently cloned in pETM-10 *E. coli* expression vector permitting the introduction of an N-terminal hexa-histidine tag. To express PdhR in mammalian cells, the *E. coli* PdhR gene sequence was codon optimized for mammalian expression and custom synthesized from GeneArt Gene Synthesis (Life technologies). This was subsequently cloned in 30 different FRET backbone plasmids (Piljic et al., 2011) between *Age*I and *Mlu*I restriction sites.

For generation of the mouse reporter PYRATES line, the FRET backbone with PdhR gene sequence from F41 vector was cloned into the pCKI vector, a modified pCAGGS (CMV-enhancer, beta-actin promoter, beta-globin polyA) ubiquitous expression vector (Niwa et al., 1991). To this end, the gene cassette was initially excised with *NheI* and *BamH*I followed by Klenow mediated filling in the 5' overhangs to generate blunt ends. This DNA fragment was introduced into *EcoRV* site of the pCKI vector. Orientation and sequence of the insert was verified by DNA sequencing.

Expression and Purification of mTurquoise-PdhR-cp173 Venus Protein

For the production of mTurquoise-PdhR-cp173Venus protein, *E. coli* expression strain BL21 DE3 was transformed with the pETM-10 plasmid containing the gene cassette. Transformed bacteria were grown in LB+kanamycin medium at 37°C until the cell density reached an O.D of 0.6. Cultures were transferred to 16°C and were induced overnight with IPTG at a final concentration of 0.5mM following which the cultures were centrifuged at 6000 rpm, 4°C. The cell pellets were resuspended in 30 ml of 20mM Tris-HCL, pH 7.6, lysed by sonication and centrifuged to obtain a cell lysate, which was incubated with equilibrated Ni-NTA beads (Amersham). The beads were then washed with 200ml of 20mM Tris, pH 7.6, 25mM imidazole and the protein eluted with 10ml of 20mM Tris, pH 7.6, 300mM imidazole. The purified protein was dialyzed against 20mM Tris, pH 7.6 and was used for in vitro FRET measurements and isothermal titration calorimetry.

In Vitro FRET Measurements

Recombinant mTurquoise-PdhR-cp173 Venus protein was excited in the presence or absence of ligands at 430nm and the fluorescence emission recorded from 460-600nm on a fluorescence microplate reader (BioTek). Ligands were added to the protein at different concentrations and incubated for 10 minutes in 20mM Tris, pH 7.6 before measurements. To study the effect of pH on the FRET response, the recombinant mTurquoise-PdhR-cp173 Venus protein was incubated for 30 minutes in the respective pH buffer (pH 7-8) and pyruvate was titrated in the same buffer and measurements recorded. FRET donor (mTurquoise) emission intensity was measured at 475nm and FRET acceptor (cp173 Venus) emission intensity was measured at 525nm. Ratio (R) of FRET

acceptor emission intensity to FRET donor emission intensity was calculated before (R_o) and after (R_f) ligand addition and the % ratio change ((R_f - R_o)/ R_o) was obtained and plotted.

Isothermal Titration Calorimetry

Pyruvate binding to recombinant mTurquoise-PdhR-cp173 Venus protein was studied by using a VP-ITC calorimeter (Microcal). The purified protein was dialyzed against 20mM Tris , pH 7.4 (ITC buffer) overnight with five changes of the buffer. Titrations were performed at 25°C by stepwise addition of 1mM pyruvate solution in ITC buffer to the 60μM protein solution in the cell. ITC data were corrected for the dilution heat and analysed using MicroCal Origin™ software package.

Cell Culture and Transfection

HeLa Kyoto were maintained and passaged in DMEM medium (Gibco) containing 4.5gL^{-1} glucose, 2mM glutamine, penicillin and streptomycin (100 U ml^{-1}) (Invitrogen, Life technologies) and supplemented with 10% fetal bovine serum (Gibco). For imaging, cells were seeded in 35mm MatTek chamber dishes with glass bottom (MatTek Corporation) and were transfected the following day with Fugene HD transfection reagent based on manufacturer's instructions. Cells were then incubated for 24 hours after which the medium was removed and replaced with imaging medium containing 20mM HEPES, pH 7.4, 115mM NaCl, 1.2mM $CaCl_2$, 1.2mM $MgCl_2$, and 1.2mM K_2HPO_4. Cells were incubated in the imaging medium for 10 minutes before imaging.

Two-Photon Microscopy

Imaging was performed with a LSM780 laser-scanning microscope (Zeiss). Samples were excited at 960nm with a Ti:Sapphire laser (Chameleon-Ultra, Coherent) through a 20x Plan-Apochromat objective (numerical aperture 0.8). A Z-stack of 6 planes with a spacing of 12μm was scanned every 10 minutes Zen software (Zeiss) was used to record all data in 12bit, 512 x 512 square pixels at 1.38μm/pixel. Mean intensity projections on all representative Z-planes were used for further analysis. Kymographs were generated in Fiji (Schindelin et al., 2012). To this end, a 10μm Gaussian filter was applied to mean intensity projections and the intensity along a line (width 21 pixels), placed from the posterior to the anterior PSM, was quantified and plotted for the entire time-series. Kymographs were then subjected to a 10μm Gaussian filter and the "Fire" lookup table was applied.

FRET Imaging and Image Processing

HeLa cells were imaged on a Leica SP2-AOBS confocal microscope (Leica Microsystems) with a 40x/1.4 oil immersion objective. Cells were excited with 405nm laser with the donor emission taken from 475 to 510nm and the acceptor emission taken from 520 to 540nm. All measurements were done at RT with the imaging settings kept constant. The pinhole was fully opened and a time series of 8 bit images were acquired with a line averaging of 2. A total of 30 scans were acquired in an experiment with the time interval between successive scans being kept constant at 10 seconds. Pyruvate was added at a final concentration of 10mM after the end of 15th scan. Image processing was done with FluoQ macro (Stein et al., 2013) using the following settings: The average of an interactively selected background ROI was used to subtract the background in each image. Images were smoothed using the median filter with a radius size of 2. The 'Triangle' thresholding method was used to threshold images and remove low value background pixels. A ratio image (FRET acceptor emission / FRET donor emission) was computed and the mean of segmented cells followed over time. For each time-trace an average was calculated before (R_o) and after (R_f) ligand addition and the % ratio change ((R_f - R_o)/ R_o) was computed using the R programming language and plotted using the ggplot2 package.

Mouse PSM 2-D ex vivo cultures expressing PYRATES were imaged on a LSM780 laser-scanning microscope (Zeiss) with a 20x/0.8 Plan-Apochromat objective. Samples were excited with a 405nm laser and mTurquoise and cp173 Venus fluorescence emission was collected from 470 to 510nm and from 520 to 540nm respectively. Images were acquired at an interval of 10 minutes. All measurements were done at 37°C, 5% $C0_2$.

Image processing was done in Fiji, the FluoQ macro (Stein et al., 2013) was used to generate FRET ratio images. A 360° radial reslice function (Fiji) from center to periphery was applied to the FRET ratio image. The median average projection of the stacks was used to quantify the average FRET ratio from center to periphery (Fiji).

QUANTIFICATION AND STATISTICAL ANALYSIS

Statistical parameters including sample sizes, the statistical test used and statistical significance are reported in the Figures and the Figure Legends. Unless otherwise indicated, values are reported as mean ± SDM. p-Values for simple pair-wise comparisons were performed using a paired two-tailed Student's t-test. Data is judged to be statistically significant when $p < 0.05$. P values for each experiment are also included in the Figures and associated Figure legends. Statistical analysis was performed using GraphPad PRISM 7 software.

Developmental Cell, Volume *40*

Supplemental Information

Spatiotemporal Analysis of a Glycolytic Activity

Gradient Linked to Mouse Embryo

Mesoderm Development

Vinay Bulusu, Nicole Prior, Marteinn T. Snaebjornsson, Andreas Kuehne, Katharina F. Sonnen, Jana Kress, Frank Stein, Carsten Schultz, Uwe Sauer, and Alexander Aulehla

Inventory of Supplemental Materials

Figure S1, related to Figure 1;
Figure S2, related to Figure 2;
Figure S3, related to Figure 3;
Figure S4, related to Figure 4;
Figure S5, related to Figure 5;
Table S1, related to Figure 1;
Movie S1 legend, related to Figure 5.

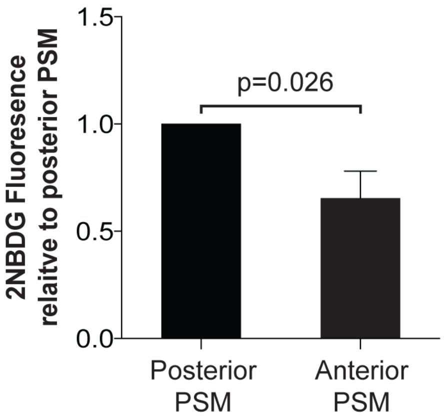

Figure S1 related to Figure 1.

Quantification of 2-NBDG fluorescence across the PSM reveals increased glucose uptake in posterior PSM after 21 hours *in vitro* culture. n=5, mean ± SDM.

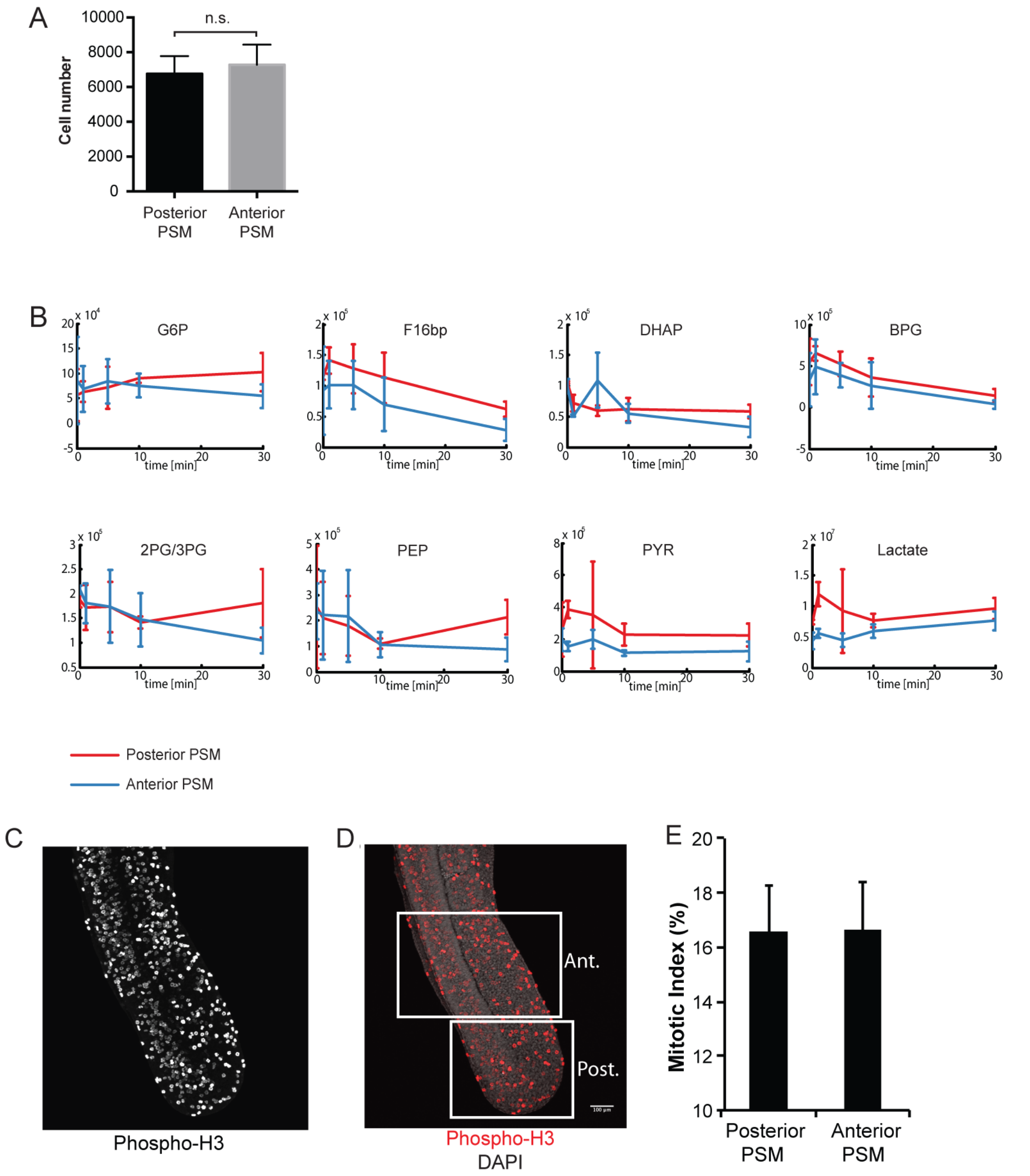

Figure S2 related to Figure 2.
Metabolite pool sizes remain constant between posterior and anterior PSM over time. (A) The number of cells counted in individual posterior and anterior PSM samples is not significantly different, n=14, mean ± SDM. (B) Summed raw intensities as a measure of intracellular pool size over the time course, n=3, mean ± SDM. Analysis of mitotic index reveals no significant difference between the anterior and posterior of mouse PSM. Embryonic tails (E10.5) were fixed directly after dissection, mitotic cells were detected using anti-Phospho-H3 antibodies (C) and counterstained with DAPI (merged in D). (E) Quantification of percentage of cells in M-phase in anterior compared to posterior PSM (N=5, mean ± SDM).

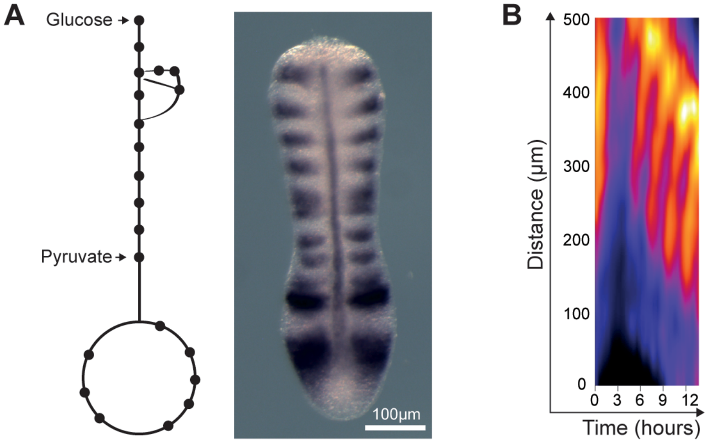

Figure S3 related to Figure 3.
Control experiment in which samples were cultured for 13 hours in medium containing 0.5mM Glucose and 10mM Pyruvate (in DMEM/F12).
(A) Whole-Mount mRNA ISH for *Lfng*, *Shh* and *Uncx4.1* following 13 hours culture is indistinguishable from results obtained in control medium (DMEM/F12=0.5mM Glucose), see Figure 3A
(B) Kymograph depicting activity of LuVeLu reporter activity within the PSM (distance from tail bud, posterior, in micrometer). Explants cultured in 0.5mM glucose + 10mM Pyruvate (in DMEM/F12) displayed regular cyclic activity of LuVeLu and segmentation throughout culture (indistinguishable from results obtained in control medium, DMEM/F12+0.5mM Glucose, see Figure 3C). n=3

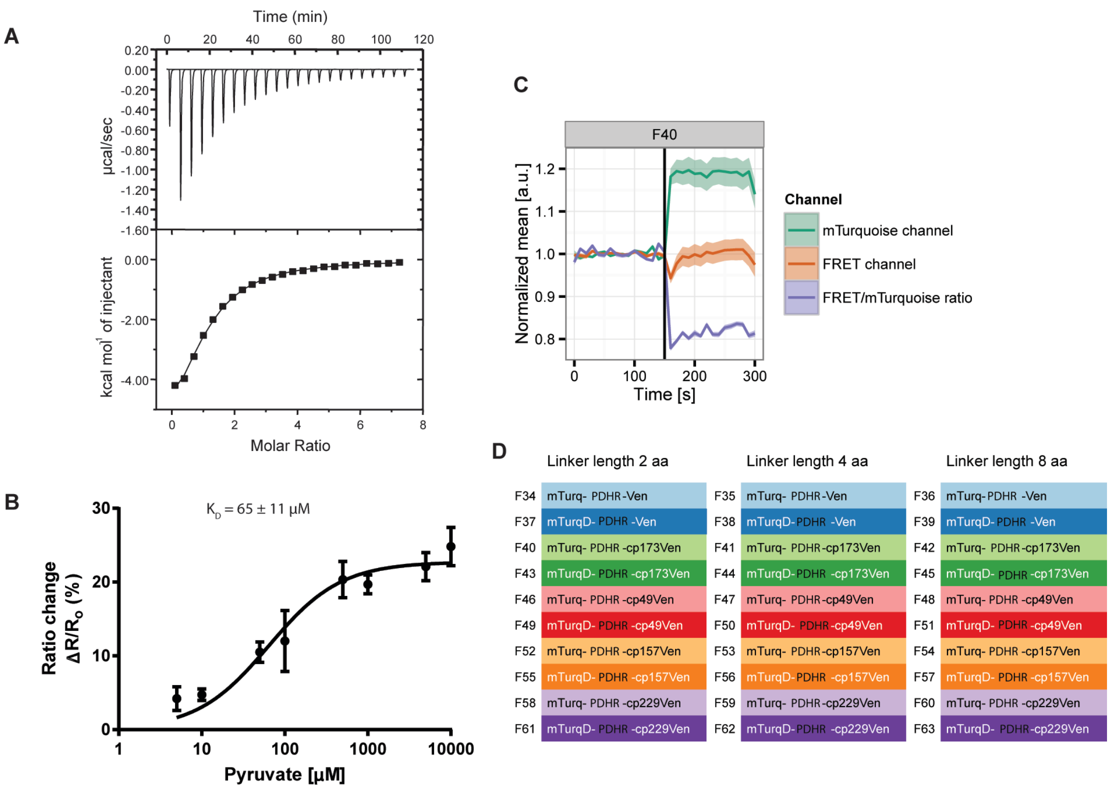

Figure S4 related to Figure 4.
(A) Pyruvate binding analysis with recombinant mTurquoise-PdhR-cp173-Venusd protein by isothermal titration calorimetry (ITC). Shown are the raw calorimetric signals obtained during the stepwise addition of pyruvate to the protein solution (60 µM) in the ITC calorimeter. The fit of the data using MicroCal Origin™ software package shows a K_d of 21 µM. (B) Pyruvate binding analysis with recombinant mTurquoise-PdhR-cp173-Venusd protein by in vitro FRET analysis. Data were fitted to a one site binding equation $Y = B_{max}*X/(K_D + [X])$, where X is the pyruvate concentration, B_{max} is the maximum specific binding and K_D is the dissociation constant using Graph pad Prism software. (C) Quantification of PYRATES-FRET signals before and after pyruvate addition in HeLa cells. Fluorescence quantification of donor and acceptor channels (mean ± SEM) before and after addition of 20mM Pyruvate (indicated by black bar). After addition of pyruvate, donor/mTurquoise fluorescence emission increases and acceptor/Venus fluorescence decreases. Overall, the Venus/mTurquoise ratio decreases upon addition of Pyruvate. The data is representative of 28 cell traces. (D) Library design for FRET optimization. Constructs with different mTurquoise (mTurq) and Venus (Ven) variants and different linker lengths (2, 4 or 8 amino acids (aa)) were used for FRET optimization.

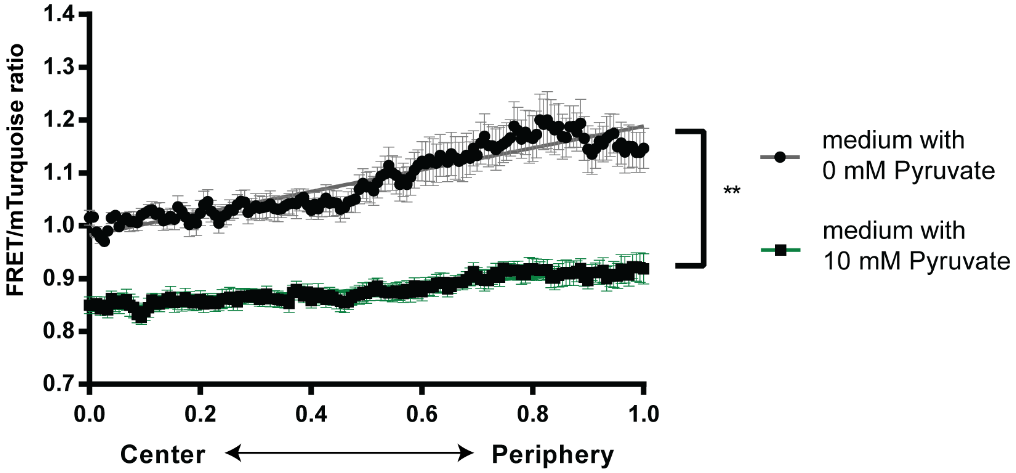

Figure S5 related to Figure 5.

FRET/mTurquoise ratio gradients from center to periphery of 2D *ex vivo* cultures (cultured for 18h) before (grey) and 2h after (green) addition of 10mM pyruvate to the control medium. Addition of exogenous pyruvate leads to a ~16% drop in mean FRET/mTurquoise ratio. The slopes of gradient before and after addition of exogenous pyruvate are significantly different. $^{**}p < 0.001$, analysis of covariance (ANCOVA) test of linear regression lines. Data is mean ± SEM, n=10 samples from N=3 independent experiments.

Table S1 related to Figure 1. Mouse embryo *In Situ* mRNA hybridization screen for genes coding for glucose carrier and metabolic enzymes.
List of 113 genes whose expression was analyzed in 10.5dpc mouse embryos. The 21 genes that were found to have elevated expression in the posterior PSM are shown in bold, genes without detectable expression are shown in red.

Glycolysis
Glucose transporter 1 (GLUT1)
Glucose transporter 3 (GLUT3)
Hexokinase I (Hk1)
Hexokinase II (Hk2)
Hexokinase III (Hk3)
Glucokinase (Gck)
ADP dep. glucokinase (Adpgk)
Glucose phosphate isomerase 1 (Gpi1)
Phosphofruktokinase Platelet (Pfkp)
Phosphofruktokinase Liver (Pfkl)
Phosphofruktokinase Muscle (Pfkm)
Aldolase A (Aldoa)
Aldolase B (Aldob)
Aldolase C (Aldoc)
Triose phosphate isomerase 1 (Tpi1)
Glyceraldehyde-3P dehydrogenase (Gapdh)
Phosphoglycerate kinase 1 (Pgk1)
Phosphoglycerate kinase 2 (Pgk2)
Phosphoglycerate mutase 1 (Pgam1)
Phosphoglycerate mutase 2 (Pgam2)
Enolase 1 (Eno1)
Enolase 2 (Eno2)
Enolase 3 (Eno3)
Enolase 1B, retrotransposed (Eno1b)
Pyruvate kinase muscle (Pkm)
Lactate dehydrogenase A (Ldha)
Lactate dehydrogenase B (Ldhb)
Lactate dehydrogenase C (Ldhc)
Glucose transporter 2 (GLUT2)
Glucose transporter 4 (GLUT4)
Glucose transporter 5 (GLUT5)
Glucose transporter 6 (GLUT6)
GAPDH spermatogenic (Gapdhs)
Pyruvate kinase liver & red blood cell (Pklr)

Gluconeogenesis
Fructose bisphosphatase 1 (Fbp1)
Fructose bisphosphatase 2 (Fbp2)
Phosphoenolpyruvate carboxykinase 1 (Pck1)
Phosphoenolpyruvate carboxykinase 2 (Pck2)

TCA, PDH
Pyruvate dehydrogenase component X (Pdhx)
Pyruvate dehydrogenase E1 α1 (Pdha1)
Pyruvate dehydrogenase E1 α2 (Pdha2)
Pyruvate dehydrogenase β (Pdhb)
Pyruvate Carboxylase (Pcx)
Dihydrolipoamide S-acetyltransf. (Dlat)
Dihydrolipoamide dehydrogenase (Dld)
Citrate synthase (Cs)
Citrate synthase like (Csl)
ATP citrate lyase (Acly)
Aconitase 1 (Aco1)
Aconitase 2, mitochondrial (Aco2)
Isocitrate dehydrogenase 1 (Idh1)
Isocitrate dehydrogenase 2 (Idh2)
Isocitrate dehydrogenase 3β (Idh3b)
Isocitrate dehydrogenase 3γ (Idh3g)
Oxoglutarate dehydrogenase (Ogdh)
Oxoglutarate dehydrogenase like (Ogdhl)
Dihydrolipoamide S-succinyltransferase (Dlst)
Succinate-CoA ligase α, GDP (Suclg1)
Succinate-CoA ligase β, GDP (Suclg2)
Succinate-CoA ligase β, ADP (Sucla2)
Succinate dehydrogenase A (Sdha)
Succinate dehydrogenase B (Sdhb)
Succinate dehydrogenase C (Sdhc)
Fumarate hydratase 1 (Fh1)
Malate dehydrogenase 1 (Mdh1)
Malate dehydrogenase 2 (Mdh2)
Isocitrate dehydrogenase 3α (Idh3a)

Acetate metabolism
Aldehyde dehydrogenase 1A3 (Aldh1a3)
Aldehyde dehydrogenase 1B1 (Aldh1b1)
Aldehyde dehydrogenase 2, mit. (Aldh2)
Aldehyde dehydrogenase 3A1 (Aldh3a1)
Aldehyde dehydrogenase 3A2 (Aldh3a2)
Aldehyde dehydrogenase 3B1 (Aldh3b1)
Aldehyde dehydrogenase 7A1 (Aldh7a1)
Aldehyde dehydrogenase 9A1 (Aldh9a1)
Acyl-CoA synthase 1 (Acss1)
Acyl-CoA synthase 2 (Acss2)

Pentose phosphate pathway
G6P dehydrogenase X (G6pdx)
H6P dehydrogenase (H6pd)
6-phosphate gluconolactonase (Pgls)
Phosphogluconate dehydrog. (Pgd)
Ribulose-5P-3-epimerase (Rpe)
Ribose-5P isomerase A (Rpla)
Transketolase (Tkt)
Transketolase like 1 (Tktl1)
Transketolase like 2 (Tktl2)
Transaldolase 1 (Taldo1)
Ribokinase (Rbks)
Phosphoglucomutase 1 (Pgm1)
Phosphoglucomutase 2 (Pgm2)
Phosphoribosyl pyrophatase synthetase 1 (Prps1)
Phosphoribosyl pyrophatase synthetase 2 (Prps2)
2-deoxyribose-5P aldolase homolog (Dera)
G6P dehydrogenase 2 (G6pd2)

Galactose metabolism
Aldo-keto reductase 1B3 (Akr1b3)
Aldo-keto reductase 1B7 (Akr1b7)
Aldo-keto reductase 1B8 (Akr1b8)
Galactosidase α (Gla)
Galactose mutarotase (Galm)
Galaktokinase 2 (Galk2)
Galactose-1P uridyl transf. (Galt)
UDP-glucose pyrophosphorylase 2 (Ugp2)
Glucose-6P, catalytic (G6pc)
Lactase (Lct)
Galactosidase β1 (Glb1)
Glucosidase α, acid (Gaa)
Glucosidase α, neutral C (Ganc)
UDP-Gal:βGlcNac β 1,4-Galactosyltransf. 1 (B4galt1)
UDP-Gal:βGlcNac β 1,4-Galactosyltransf. 2 (B4galt2)
Maltase-Glucoamylase (Mgam)
Galactokinase 1 (Galk1)
Lactalbumin α (Lalba)
Galactose-4-epimerase, UDP (Gale)

Metabolic pathway:	Total genes screened:	Detected in the embryo:	Detected in the PSM:	Glycolytic PSM expresion Pattern:
All pathways	113 genes	99 genes	79 genes	21/79 (27%)
Glycolysis	34 genes	28 genes	24 genes	17/24 (71%)
TCA, PDH	29 genes	28 genes	22 genes	3/22 (14%)
PPP	17 genes	16 genes	13 genes	0/13 (0%)
Acetate metabolism	10 genes	10 genes	6 genes	0/6 (0%)
Gluconeogenesis	4 genes	4 genes	1 gene	0/1 (0%)
Galactose metabolism	19 genes	15 genes	14 genes	1/14 (0.07%)

Movie S1 legend, related to Figure 5. Real-time imaging of FRET-pyruvate sensor reporter PYRATES in 2-D *ex vivo* mesoderm segmentation assay. Tail bud mesoderm derived from a 10.5dpc mouse embryo heterozygous for the PYRATES transgene was used for 2-D *ex vivo* culture. Real-time imaging reveals the formation of a FRET ratio gradient from center to periphery during the course of *ex vivo* culture, which corresponds to cells with posterior and anterior PSM identify, respectively. A quantification of this movie is shown as kymograph in Fig5F.
